# Intrinsically disordered protein droplet-enhanced oligonucleotide assembly enables rapid oligonucleotide-to-protein expression

**DOI:** 10.1093/nar/gkag431

**Published:** 2026-05-04

**Authors:** Taiji Ueno, Yoshihiro Minagawa, Yasushi Okada, Hiroyuki Noji

**Affiliations:** Department of Applied Chemistry, Graduate School of Engineering, The University of Tokyo, Tokyo 113-0033, Japan; Department of Applied Chemistry, Graduate School of Engineering, The University of Tokyo, Tokyo 113-0033, Japan; Department of Physics, and Universal Biology Institute (UBI), Graduate School of Science, The University of Tokyo, Hongo, Tokyo 113-0033, Japan; Laboratory for Cell Polarity Regulation, RIKEN Center for Biosystems Dynamics Research, Kobe, Hyogo 650-0047, Japan; Department of Cell Biology, Graduate School of Medicine, The University of Tokyo, Hongo, Tokyo 113-8654, Japan; International Research Center for Neurointelligence (WPI-IRCN), The University of Tokyo, Hongo, Tokyo 113-0033, Japan; Department of Applied Chemistry, Graduate School of Engineering, The University of Tokyo, Tokyo 113-0033, Japan; Research Institute of Planetary Health (RIPH), The University of Tokyo, Tokyo 108-0074, Japan

## Abstract

Recent advances in *in silico* protein design and bioinformatics have enabled the rapid generation of candidate sequences for functional proteins. However, experimental validation remains a bottleneck, largely due to time-consuming DNA assembly and cell-based cloning processes. Technologies that reduce the time required to convert synthetic oligonucleotides (oligos) into expressed proteins are therefore of considerable interest. Here, we demonstrate that phase-separated droplets formed by the intrinsically disordered protein (Ddx4^N1^) concentrate both oligos and ligation enzymes, enabling efficient oligo assembly at nanomolar to sub-nanomolar concentrations that are typically inaccessible to conventional ligation-based methods. The assembled products can be directly introduced into femtoliter-scale microreactors for digital cell-free gene expression, allowing protein expression from single assembled DNA molecules without polymerase chain reaction amplification or cellular cloning. Simultaneous expression of two distinct proteins from separately assembled DNA templates in a one-pot reaction was also demonstrated. The complete workflow—from oligo assembly to detectable protein expression—can be performed within half a day. While further development will be required to enhance reaction parallelization and enable systematic retrieval of sequence information from expressed products, this amplification-free, low-input system establishes a technical foundation for integrating oligo-pool-based gene assembly with digital protein prototyping platforms.

## Graphical Abstract

**Graphical Abstract:**
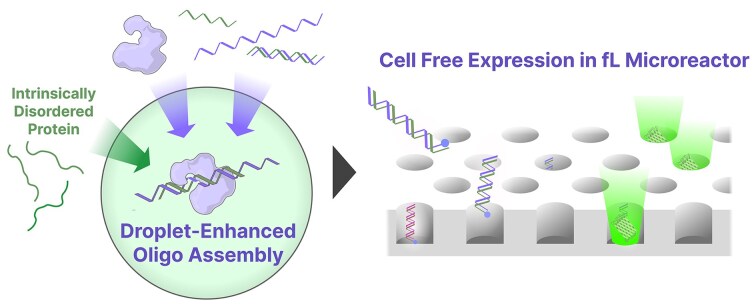

## Introduction

Deep learning-based protein design technologies have made remarkable progress, enabling the rapid generation of protein sequences with desired functions [1–3]. Enzyme discovery methods based on bioinformatic analysis have also advanced significantly, allowing the identification of functional protein sequences [4]. However, the downstream experimental steps—such as preparing DNA encoding the designed proteins and expressing the genes—remain costly and time-consuming, thus constituting a major bottleneck in the workflow [2, 5, 6]

Current protein prototyping methodologies typically begin with assembling DNA from chemically synthesized single stranded oligonucleotides (oligos) of limited length (200–300 nucleotides) [7]. While protein-coding DNA is typically purchased directly from commercial vendors, the prohibitive costs associated with large-scale screening make the use of oligo pools a more advantageous and cost-effective alternative. While commercial gene synthesis costs approximately $170 USD per kilobase, laboratory-based assembly from oligo pools can generate a 1kbp construct for an estimated $1–7 USD per gene when sufficient parallelization is implemented (Supplementary Fig. S1 and Supplementary Table S4). Thus, these oligo pools constitute a powerful methodology for large-scale prototyping.

Previous approaches to obtaining large scale DNA libraries with oligo pools have focused on increasing parallelization [8–13]. For example, DropSynth, a highly parallelized oligo assembly method using water-in-oil emulsions, employs DNA barcoding to selectively compartmentalize sets of oligos, thereby enabling massively parallel DNA assembly [11–13]. Similarly, Golden Gate Assembly has been developed as a more advanced DNA cloning strategy [14], offering improved compatibility with parallel, one-pot oligo assembly compared with polymerase cycling assembly [15]. This method enables one-pot assembly of ~10 protein-coding DNA sequences and, when combined with a polymerase chain reaction (PCR)-based sub-pooling strategy, has the potential to generate on the order of ~1000 protein-coding genes in parallel [15].

While these approaches are promising as next-generation platforms for protein prototyping, further improvements are required to accelerate the transition from design to functional analysis and integrate the entire process with more high-throughput protein expression systems such as cell-free expression. Although oligo pool synthesis has reduced costs by minimizing individual oligo yields, this low starting material often complicates downstream assembly [7]. For instance, while recently developed methods like Sidewinder employ three-way junctions to enable highly accurate parallel assembly, they have not yet achieved direct assembly from oligo pools because their complex oligo interactions remain highly susceptible to extremely low effective concentrations [16]. As a result, current protocols typically rely on pre-amplification prior to assembly [7, 10, 15, 17], a process that not only introduces sequence-dependent biases but also necessitates the addition of primer-binding sites. These flanking sequences not only constrain the effective protein-coding length but also require subsequent, labor-intensive enzymatic removal. Furthermore, while cell-free protein synthesis enables high-throughput functional screening of enzymes directly from DNA templates [18–22], the upstream oligo assembly process has yet to be integrated into these screening platforms. Therefore, technologies that facilitate rapid DNA assembly and its direct coupling with cell-free gene expression are urgently required.

Highly parallelized systems for high-throughput protein prototyping inherently require assembly technologies capable of operating from extremely small quantities of oligos, particularly when derived from large oligo pools. Motivated by this requirement, we developed a simple and rapid protocol for oligo assembly that enables protein expression directly from low-input oligos without PCR amplification. We utilize phase-separated droplets formed by an intrinsically disordered protein (IDP), Ddx4^N1^—the N-terminal disordered region of the DEAD-box 4 helicase Ddx4. Ddx4^N1^ is referred to as Ddx4 for simplicity throughout this study. Ddx4 droplets have been reported to enrich oligos [23, 24], and we exploit this oligo-enrichment capability to enhance assembly efficiency at low concentrations. By integrating this Ddx4 droplet-enhanced assembly method with digital cell-free gene expression, we establish and evaluate a low-input oligo-to-protein workflow that addresses a key technical requirement for future high-throughput protein prototyping systems.

## Materials and methods

### Protein expression and purification

The Ddx4^N1^ construct was cloned into a *pET* vector containing TwinStrepTag and SUMO tags for purification purposes. The complete amino acid sequence is provided in Supplementary Table S2. The plasmid construct design is shown in Supplementary Fig. S20. Competent Rosetta™ 2(DE3) *pLysS* Singles cells (Sigma–Aldrich) were transformed with the plasmid and cultured in LB medium [Tryptone (Nacalai Tesuque) 1% (w/v), Bacto Yeast Extract (Gibco) 0.5% (w/v), Sodium chloride (Fujifilm Wako) 1% (w/v)] supplemented with ampicillin (100 μg/ml) at 37°C with shaking at 140 rpm. Protein expression was induced with 1 mM IPTG (Fujifilm Wako) at OD_500_ = 0.7, followed by overnight incubation at 20°C with shaking at 140 rpm. Bacterial pellets were resuspended in Buffer A (50 mM Tris–HCl, pH 8.0, 500 mM NaCl, 5 mM dithiothreitol (DTT)) supplemented with Roche Protease Inhibitor Complete Mini. Cells were lysed by sonication and clarified by ultracentrifugation at 50 000 rpm for 15 min at 4°C. The supernatant was applied to a Strep–Tactin Superflow Plus column (QIAGEN) pre-equilibrated with Buffer A. After binding for 2 h at 4°C, the column was washed extensively with Buffer A. SUMO tag cleavage was performed on column using SUMO protease (final concentration 5 µM, purified in our lab) in SUMO Protease Buffer overnight at 4°C. The eluate was subsequently applied to a Ni-NTA column (QIAGEN) to remove un-cleaved fusion protein and SUMO protease. Flow-through fractions containing purified Ddx4 were concentrated using Amicon Ultra centrifugal filters (MWCO 3K, Millipore Sigma) and buffer-exchanged into Storage Buffer (20 mM Tris–HCl, pH 8.0, 300 mM NaCl, 5 mM TCEP). Protein purity was assessed by sodium dodecyl sulfate-polyacrylamide gel electrophoresis with Coomassie blue staining (Nacalai Tesque). Final protein concentration was determined by UV absorbance at 280 nm using an extinction coefficient of 24 000 M^−1^cm^−1^. The purified protein was stored at −80°C at ~100 μM concentration. For phase separation experiments, purified Ddx4 was buffer exchanged into Storage Buffer containing 30 mM NaCl or pure water using Amicon Ultra centrifugal filters (MWCO 3K, 0.5 ml). The protein was concentrated to 200 μM and stored at 4°C for up to 3 days.

### Oligo design and DNA labeling

Fluorescently labeled oligos were synthesized commercially (FASMAC, reverse-phase column purification). Four oligo systems were designed: System 1 (9-oligo assembly), System 2 (mNeonGreen expression), System 3 (extended mNeonGreen expression), and System 4 (mScarlet expression). The detailed sequences are included in the Supplementary Table S3. Unlabeled DNA was fluorescently labeled using Label IT Fluorescein reagent (Mirus Bio) and purified using G50 Microspin columns (Cytiva) for partitioning experiments.

### Microscopic observation of phase-separated droplets

For the experiment to measure the partition coefficient (PC), Ddx4 phase separation was induced in the presence of various fluorescently labeled DNAs or Alexa647 modified T4 Ligase and PNK (polynucleotide kinase) on glass coated with CYTOP CTL-816AP fluoropolymer (Asahi Glass). For annealing experiments using FRET, phase-separated Ddx4 droplets (70 μM final concentration) were formed in 384-well plates (Greiner) in the presence of 0.5 μM 80-nt 3′ FAM oligo and 1× T4 Ligase Buffer (included in Takara T4 Ligase set). Either 20-nt 5′ Cy5 (annealing condition) or 20-nt 5′ Cy5-nonannealing (nonannealing control) was added to a final concentration of 0.5 μM. Confocal microscopy was performed using a Leica TCS SP8 system with a 100× oil immersion objective. FRET efficiency was calculated as: *F*_A_ / (*F*_A_ + γ*F*_D_), where *F*_A_ and *F*_D_ represent fluorescence intensities in the donor and acceptor channels. The procedure for calculating γ is described in the Supplementary Materials. Ten droplets were analyzed per condition. The used objective lens was HC PL APO 100×/1.47 OIL CORR TIRF. Fluorescence detection settings: Alexa647 (T4 Ligase or PNK) and Cy5 detection: λ_ex_ 640 nm, λ_em_ 670–760 nm. FAM and fluorescein detection (F_D_): λ_ex_ 490 nm, λ_em_ 504–560 nm; FRET between FAM and Cy5 detection (F_A_): λ_ex_ 490 nm, λ_em_ 658–709 nm. For measuring PC of double stranded DNA constructed by Cy5 and FAM-labeled two DNAs, Cy5 measurement was used. FLIM experiments were performed using a Leica SP8 FALCON confocal microscope system (Leica Microsystems). The experimental setup, excluding microscopy settings, was identical to that used for PC measurements. An HC PL APO CS2 93×/1.30 GLYC objective lens was used for all observations. For excitation, a 440-nm pulsed laser operating at a repetition rate of 40 MHz was utilized. Fluorescence emission was captured in the spectral range of 505–600 nm. To ensure high statistical precision for lifetime estimation, photon accumulation was continued until the maximum photon count per pixel reached 10 000. The acquired fluorescence decay curves were analyzed by a single-exponential decay model using the “n-Exponential Tail Fit” within the LAS X software suite.

### Oligo assembly and quantitative PCR analysis

Oligo assembly was performed in 20 μl reactions containing Ddx4 (70 μM), oligo mixture (25 nM–250 pM final concentration of each oligo), 1× T4 DNA Ligase Buffer contained in Takara T4 Ligase set, Takara T4 polynucleotide kinase (10 U, 0.5 U/μl final concentration), and Takara T4 DNA ligase (350 U, 17.5 U/μl final concentration). The oligo set used was Oligo Assembly System 1 in Supplementary Table S2. Reactions were incubated at 16°C for 1 h, followed by enzyme inactivation at 65°C for 15 min. Control reactions without Ddx4 were performed under identical conditions.

Oligo assembly efficiency was quantified by TaqMan quantitative PCR (qPCR) using specific primers and hydrolysis probes designed to amplify across ligation junctions (sequences in Supplementary Table S2). qPCR was performed in 20 μl reactions containing forward and reverse primers (each at 0.1 µM final concentration), a TaqMan probe (0.1 µM final concentration), 1× Ex Taq buffer supplied with the TaKaRa Ex Taq HS polymerase kit, Takara Ex Taq HS DNA polymerase (5 U per reaction), and dNTPs included in the kit (0.2 mM each, final concentration). Two microliters of the assembled products, diluted 1:100 in nuclease-free water, were added to each 20 μl qPCR reaction as template. Reactions were run on an Applied Biosystems StepOne real-time PCR system (Thermo Fisher Scientific) with the following cycling conditions: (initial denaturation at 95°C for 2 min, followed by) 60 cycles of 98°C for 10 s and 72°C for 10 s for short templates, or 40 cycles of 98°C for 10 s and 60°C for 10 s for protein expression templates. Fluorescence was recorded during the annealing/extension step. For quantification of assembly efficiency, three independent assembly reactions were analyzed (*n* = 3 biological replicates), and each sample was measured once by qPCR (no technical replicates). Ct values were determined using the automatic threshold setting of the instrument software. Standard curves generated from 10-fold serial dilutions of purified fully assembled products over 10 orders of magnitude (Supplementary Fig. S6B) could be described by DNA amount = 2922 × exp (−0.71 × Ct), corresponding to a slope of −3.24 in the Ct versus log_10_ (DNA amount) plot and an amplification efficiency of ~103% (*R*² = 0.991). No amplification was detected in no-template control reactions and un-ligated oligo control reactions (Supplementary Fig. S6C).

### Oligo assembly and sequencing analysis

Oligo assembly was performed in 20 μl reactions containing 1× T4 DNA Ligase Buffer, T4 DNA ligase (350 U, 17.5 U/μl final concentration), and T4 polynucleotide kinase (10 U, 0.5 U/μl final concentration). Oligo sets were added at final concentrations ranging from 0.5 to 500 nM depending on experimental conditions. The oligos used were from Oligo Assembly System 2 in Supplementary Table S2. For reactions with Ddx4 phase separation, Ddx4 (50 μM final concentration) were included. Reactions were incubated at 37°C for 1 h, followed by enzyme inactivation at 65°C for 15 min. Assembled products were amplified using KOD One PCR Master Mix (Toyobo) in 40 μl reactions containing 1× master mix, template DNA (4 μl of assembled product), and primers at 1 μM final concentration. The used oligos were from Oligo Assembly System 2. PCR cycling conditions were initial denaturation at 98°C for 10 s, followed by 35 cycles of 98°C for 10 s and 68°C for 30 s. PCR products were analyzed by electrophoresis on 2% agarose gels (LABTAS) in 1× TAE buffer at 125 V for 25 min, followed by SYBR Safe staining (Thermo Fisher Scientific). Target bands (~750 bp) were excised from gels and purified using GP columns (FastGene). Gel-purified PCR products were cloned using In-Fusion HD Cloning Kit (Takara). Reactions (10 μl total volume) contained 2 μl of 5× In-Fusion HD enzyme premix, 1 μl of linearized vector (8–16 ng) derived from pET vector, 1–4 μl of gel-purified insert, and nuclease-free water. Reactions were incubated according to manufacturer’s instructions.

HIT JM109 Competent Cells (Real Biotech) were transformed with 5 μl of In-Fusion reaction mixture. Cells were mixed by brief vortexing, incubated on ice for 10 min, and plated on LB agar plates containing Tryptone 1% (w/v), Bacto Yeast Extract 0.5% (w/v), Sodium chloride 1% (w/v), Agarose (Fujifilm Wako) 2% (w/v), and ampicillin (100 μg/ml, Fujifilm Wako). Plates were incubated overnight at 37°C. Each single colonies was picked and cultured in 5 ml LB medium containing ampicillin (100 μg/ml) overnight at 37°C. Plasmid DNA was isolated using miniprep kits according to standard protocols of FastGene Plasmid Mini Kit. Plasmid concentrations were measured using NanoDrop spectrophotometry. Samples with concentrations ≥18 ng/μl were used for Sanger sequencing analysis (FASMAC).

### Femtoliter reactor array device fabrication and biotin-PEG-modification

Following a previous study [25], the femtoliter reactor array devices (FRADs) were prepared using photolithography microfabrication. For the biotinylation of FRAD, the photomasks on the FRAD were not washed at the final step. Biotin-PEG-modified FRAD were prepared through silanization and PEGylation treatments. Microreactors were arranged on racks, immersed in deionized water, and degassed for 30 min. For silanization, 120 ml of deionized water was heated to 90°C with stirring, and acetic acid (0.1% v/v, 120 μl) and (3-Mercaptopropyl) trimethoxy silane (TCI) (1% v/v, 1.2 ml) were added. After 10 min of stirring to dissolve aggregates, device racks were immersed in the solution and incubated at 90°C for 2 h. Devices were then washed extensively with deionized water and dried at 110°C for 10 min. For biotinylation, Biotin-PEG3K-Maleimide was dissolved in 1M phosphate buffer (pH 7.0) to achieve 20 mg/ml concentration. Biotin-PEG3K-Maleimide solution (70 μl) was applied to each device, and devices were sandwiched with resist-coated surfaces in contact. After degassing to 0.06 MPa (repeated twice), devices were incubated for 2 h under humid conditions. Devices were then washed 10 times with deionized water, treated sequentially with acetone (5 min), fresh acetone (Nacalai, 5 min), and 2-propanol (5 min) with sonication, washed again with deionized water, and dried under nitrogen gas. Processed devices were stored in light-protected conditions.

Accordingly, the flow cells were assembled with a FRAD, a top glass with inlet and outlet holes, and using a double-sided tape (~80 μm) as a spacer [25]. The top glasses for the flow cell were precoated with CYTOP 809M to avoid the nonspecific binding of biomolecules.

### Oligo assembly and cell-free protein expression

Oligo assembly was performed in 20 μl reactions containing Ddx4 (80 μM), oligo mixture (500–0.5 nM final concentration of each oligo), 1× T4 DNA Ligase Buffer, T4 polynucleotide kinase (10 U), and T4 DNA ligase (350 U). Reactions were incubated at 37°C for 1 h. Control reactions without Ddx4 were performed under identical conditions. The used oligo set for mNeonGreen expression was Oligo Assembly System 3. The used oligo set for mScarlet expression was Oligo Assembly System 4. The used oligo set for mNeonGreen and mScarlet parallel expression was mixed set of Oligo Assembly System 3 and 4 in Supplementary Table S2. Assembled products were converted into double-stranded templates suitable for cell-free expression. Reactions (40 μl total volume) contained 1× Ex Taq HS PCR buffer (Takara), 5 mM dNTPs (Takara), 10 U Ex Taq HS polymerase (Takara), 1 μM Extension Primer, and 20 μl of oligo assembled product. The thermal cycling program consisted of initial denaturation at 95°C for 3 min, followed by annealing and extension steps at 45°C for 5 min, 55°C for 5 min, and 65°C for 5 min. Twenty microliters of double-stranded product was incubated with NEB Exonuclease I (40U, 1U/μl) in 1× reaction buffer (40 μl total volume) at 37°C for 30 min, followed by enzyme inactivation at 80°C for 20 min.

Biotinylated FRAD was incubated with streptavidin solution (pre dissolved for 1 mg/ml in PBST, Funakoshi) for 30 min at room temperature, washed extensively, and used for oligo immobilization. Templates were immobilized in biotin-PEG-modified FRAD via streptavidin interaction. Cell-free protein expression was performed using NEB PURExpress system supplemented with AlexaFluor647 (1.25 μM) and RNase inhibitor (2 U/μl). Reactions were encapsulated with fluorinated oil (AE3000 + 0.10% S386; AGC), sealed with Fomblin (Solvay) and incubated at 30°C for up to 16 h. Fluorescence imaging was performed using a Nikon TiE microscope (60× oil objective) with appropriate filter sets for AlexaFluor647, mNeonGreen, and mScarlet detection. Automated particle detection identified FRAD based on AlexaFluor647 signal. Fluorescence intensities were quantified using ImageJ and analyzed using custom Python scripts. Gaussian models were fitted to establish intensity thresholds. Multi-step filtering eliminated false positives based on intensity criteria, ROI comparison, and proximity filtering. The detailed methods of image analysis are included in the Supplementary Materials and Methods.

## Results

### Enrichment of DNA in Ddx4 droplets

**Figure 1.**
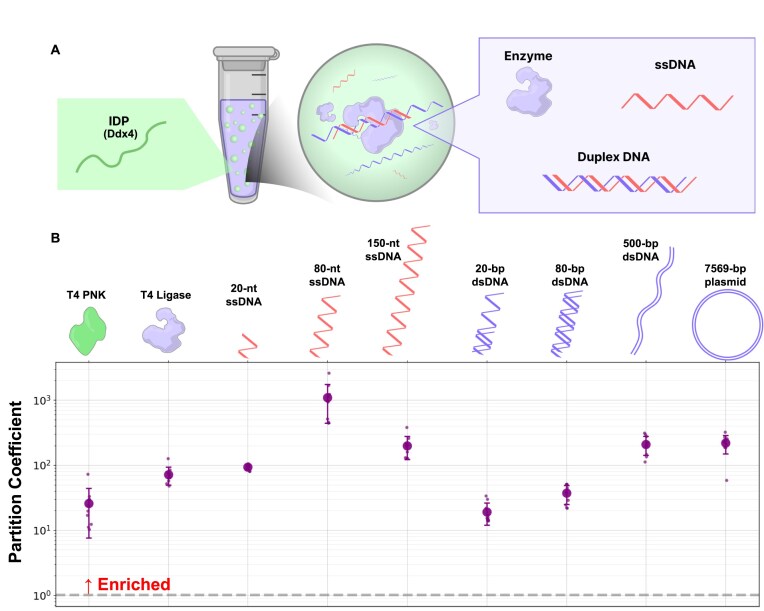
Enrichment ratio of various DNA and Ligase. (**A**) Schematic illustration of IDP (Ddx4) droplet-enhanced oligo assembly. (**B**) PCs of DNA or ligase protein labeled with fluoresce dye [Cy5 (Alexa647), FAM, Fluorescein]. PC was determined as the ratio of fluorescence intensity inside the droplet to that outside. Values greater than 1 indicate preferential enrichment in droplets. See the “Materials and methods” section for details. *N* = 10 for each condition. Each PCs was corrected based on FLIM experiments (Supplementary Fig. S2).

Motivated by the previous report that single-stranded DNAs (ssDNAs; 12–40 nt) are concentrated within Ddx4 droplets [23], we quantitatively investigated the enrichment capability of Ddx4 droplets for various types of DNAs (Fig. 1A). The PC, defined as the concentration of a target molecule inside droplets divided by that in the surrounding medium, was determined using fluorescently labeled DNAs and confocal microscopy. The measured values were corrected based on FLIM analysis, which revealed differences in the quantum yield of the fluorescent dye inside and outside the droplets (Supplementary Fig. S2). Droplets were analyzed within the first hour after DNA addition, as the oligo assembly reaction is completed within ~1 h under these conditions.

The measured PC values for ssDNA and double-stranded DNA (dsDNA) ranged from 19.1 to 1093 (Fig. 1B). The lowest PC values were observed for short DNAs: 20-nt ssDNA and 20-bp dsDNA showed PCs of 93.4 and 19.1, respectively, whereas the highest PC (1093) was obtained for 80-nt ssDNA. For dsDNA, a clear trend of increasing PC with DNA length was observed. We also examined fluorescently labeled T4 DNA ligase and T4 PNK under the same conditions, which exhibited PCs of 71.8 and 26.1, respectively (Fig. 1B). These results indicate that both DNA substrates and ligation enzymes are enriched within Ddx4 droplets, supporting the feasibility of droplet-enhanced oligo assembly.

**Figure 2.**
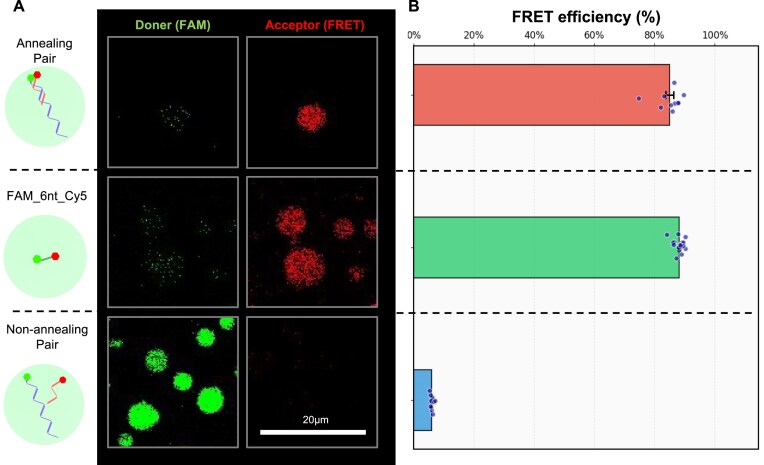
Annealing inside Ddx4 droplets. (**A**) Fluorescence image of fluorescent signal in droplets (left: donor; FAM, right: acceptor; FRET signal). (**B**) FRET efficiency of 80- and 20-nt ssDNA pair, each labeled with FAM and Cy5 (annealing pair), double-labeled 6-nt ssDNA with FAM and Cy5, or nonannealing pair of ssDNAs (nonannealing pair). *N* = 10 for each condition. The FRET efficiency was calculated as *F*_A_ / (*F*_A_ + γ*F*_D_). The procedure for calculating γ is described in the Supplementary Materials and Methods.

Notably, while a previous study reported that dsDNA longer than 20 bp is excluded from Ddx4 droplets [23], our results show efficient enrichment of dsDNA, with PC values ranging from 20 to 200. The earlier report also suggested that dsDNA tends to dissociate into single strands within Ddx4 droplets [23]. To investigate whether such dissociation occurs, we performed FRET analysis. As a model system, we prepared a pair of 20-nt and 80-nt ssDNAs that form dsDNA by annealing through a 20-nt complementary region. Each ssDNA was labeled with a fluorophore: the 20-nt strand with a donor (FAM) and the 80-nt strand with an acceptor (Cy5) (Supplementary Fig. S3). Using confocal microscopy, we measured donor and acceptor fluorescence signals (*F*_D_ and *F*_A_) inside Ddx4 droplets and calculated the FRET efficiency as *F*_A_ / (*F*_A_ + γ*F*_D_) (Fig. 2 and Supplementary Fig. S4). The procedure for calculating γ is described in the Supplementary Materials and Methods. The annealed pair showed a high FRET efficiency of 84.9%, comparable to that of a control ssDNA labeled with FAM and Cy5 at both termini (88.1%) (Supplementary Fig. S5B). A negative control pair lacking complementary sequences showed only 5.9%, confirming that enrichment alone does not enhance FRET efficiency. These results clearly demonstrate that complementary oligos remain annealed within Ddx4 droplets.

However, it should be noted that the FRET efficiency of the annealed dsDNAs gradually declined to approximately half its initial value over time (Supplementary Fig. S5). Although the signal was high immediately after mixing and enrichment in Ddx4 droplets, it monotonically decreased to 47.6% of its initial value after 9 h. The positive control retained its high FRET efficiency throughout, indicating that the observed decline was not due to photobleaching or other photochemical artifacts. While the mechanism behind this slow dissociation remains unclear, the fact that a substantial fraction of annealed oligos remain intact for several hours suggests that oligo assembly reactions are feasible within Ddx4 droplets.

### Ddx4 droplet-enhanced oligo assembly

We subsequently examined the enhancement of oligo assembly by Ddx4 droplets. Considering the application to the direct assembly from oligos without pre-amplification process, we employed direct ligation using DNA ligase—the simplest oligo assembly method—which requires only single-stranded oligos and does not depend on amplified dsDNA templates.

**Figure 3.**
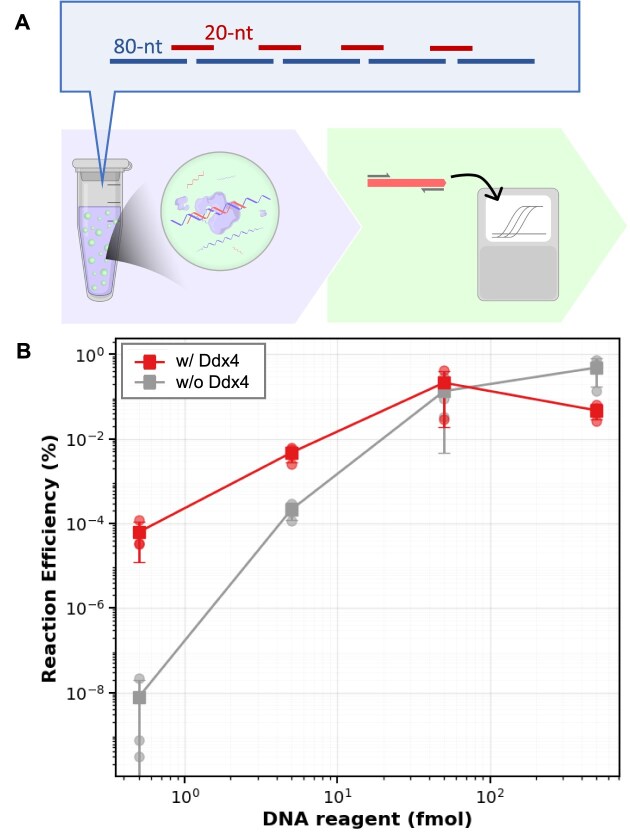
qPCR analysis of oligo assembly. (**A**) Schematic diagram showing the oligo design and qPCR analysis workflow. (**B**) Reaction efficiency versus the initial oligo concentration. Reaction efficiency was defined as assembled DNA yield divided by theoretical maximum yield estimated from the initial oligo concentration. droplet-enhanced assembly (red) and assembly w/o droplet (gray). *N* = 3.

As the initial model system, we designed a reaction using five 80-nt oligos (Fig. 3A). Each oligo was designed with 20-nt complementary regions at its termini, allowing the formation of nicked double-stranded structures upon annealing. T4 DNA ligase was then used to ligate the nicks, completing the oligo assembly. The assembly product was designed to form the totally 80-nt double stranded regions and the totally 320-nt single stranded regions (Fig. 3A). Reactions were conducted at 16°C for 1 h.

We quantified the reaction efficiency using a TaqMan probe-based qPCR assay. For the quantification, we first prepared qPCR calibration curves using fully assembled dsDNA (Supplementary Fig. S6A). We defined the reaction efficiency as the ratio of the amount of detected assembly product to the theoretical maximum assuming complete assembly of all oligos (Fig. 3B). The results clearly show that the Ddx4 droplet-enhanced oligo assembly showed enhanced efficiency in particular at low oligo concentrations; at 250 pM that corresponds to 5 fmol of oligos in total reaction mixture, the assembly efficiency was increased 22-fold. At 25 pM (0.5 fmol), the enhancement factor rose to 3.3 × 10^4^ fold. These findings show that Ddx4 droplets enable highly efficient oligo assembly even at extremely low oligo concentrations.

The reaction yield of the oligo assembly was at best only 1% under the current condition, meaning that most of oligo molecules were left not properly incorporated into the assembled product. Therefore, a possible concern is that the unincorporated oligo molecules could interfere with the qPCR quantification. To test this possibility, we performed control experiments: the serial dilutions of the reaction mixture with 250 nM oligo concentration were analyzed (Supplementary Fig. S6B). The threshold cycle (*C*_t_) values exhibited a linear correlation with dilution factors. In addition, we repeated qPCR analysis in the presence of Ddx4 droplets. The *C*_t_ values were essentially unchanged compared to those in buffer-only conditions (Supplementary Fig. S6C). Thus, it was shown that unincorporated oligo or Ddx4 droplets did not interfere with the qPCR quantification. We also ensured the specificity of qPCR by testing the mixture of oligos without complemental sequences that did not show amplification (Supplementary Fig. S6C).

### Sequence analysis of oligo assembly

To evaluate the applicability of our method for constructing functional protein-coding DNA, we extended droplet-enhanced oligo assembly to generate DNA encoding mNeonGreen, a widely used fluorescent protein (Supplementary Fig. S7A). Five 150-nt oligos were designed with 20-nt complementary overlaps at their termini to form nicked double-stranded structure upon annealing and ligation. The expected full-length product is 750 bp.

Initially, oligo assembly reactions were conducted at 16°C for 1 h using 250 nM oligos. However, no assembled product was detected (Supplementary Fig. S7B). We hypothesized that the longer oligos (150 nt versus 80 nt) are prone to form secondary structures that impeded assembly. Raising the oligo assembly temperature to 37°C successfully restored oligo assembly activity. Subsequently, ligation reactions were performed at 37°C. Using qPCR, we evaluated the assembly efficiency at lower oligo concentrations, 5 nM and 0.5 nM. At both concentrations, Ddx4 droplet markedly enhanced assembly; a 578-fold increase in assembly efficiency was observed at 5 nM compared to assembly reactions without droplets. At 0.5 nM, assembled product was undetectable without Ddx4 droplets (Supplementary Fig. S7C).

To verify the structural integrity and functionality of droplet-enhanced assembly, the assembled products were analyzed by agarose gel electrophoresis after PCR amplification. For the product prepared at higher oligo concentrations (500 and 50 nM), bands corresponding to the expected size were observed regardless of the presence of Ddx4 droplets. At lower concentrations (5 and 0.5 nM), detectable PCR products were obtained only for droplet-enhanced assembly (Supplementary Fig. S8A).

**Figure 4.**
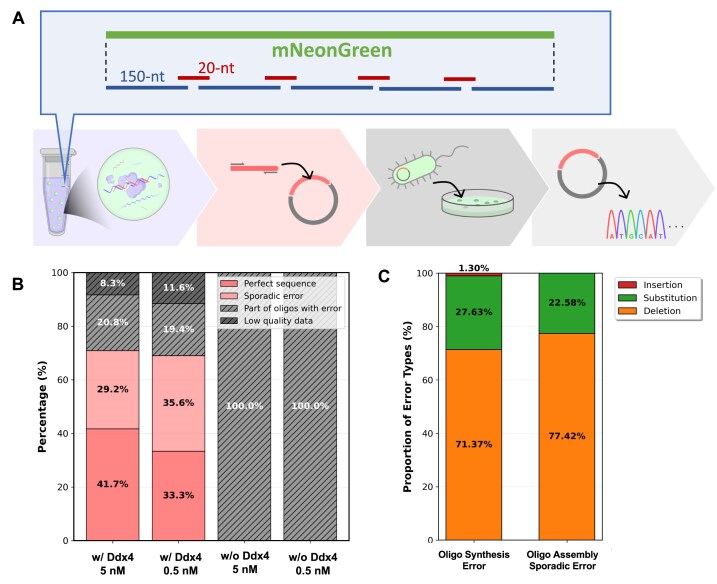
Sequencing analysis of assembled DNA. (**A**) Schematic image of experimental workflow for sequence analysis of assembled DNA. (**B**) Results of sequencing analysis. Dark red bars indicate the fraction of reads with the perfect sequence (designed one). Light red bars are the fraction of reads with sporadic errors (substitutions or deletion of 1–3 nt) that are attributable to oligo synthesis error. Light black bars indicate the fraction of reads with a large deletion. Dark black bars represent the fraction of unreadable results with low-quality signals. Cloning of droplet-enhanced assembly was repeated (*N* = 3), and 8 clones were sequenced in each experiment (24 clones in total) for droplet-enhanced assembly. Because assembly w/o droplet yielded only few colonies, 3–6 clones were sequenced. (**C**) Comparison of error types between next-generation sequencing (NGS)-detected oligo synthesis errors and sporadic errors from oligo assembly. Error types are color-coded: orange indicates deletions, green indicates substitutions, and red indicates insertions. The left panel shows oligo synthesis errors detected by NGS sequencing, while the right panel displays sporadic errors (*n* = 31) shown in panel (B).

The PCR products were gel-purified and cloned into plasmids, followed by transformation into *Escherichia coli* cells. In droplet-enhanced oligo assembly, 23.3 ± 11.5 and 15.3 ± 2.5 colonies were obtained from the 5 nM and 0.5 nM reactions, respectively (Supplementary Fig. S8B, left). In contrast, only 2.0 ± 0.0 colonies were obtained at 5 nM and 1.0 ± 1.0 colony at 0.5 nM from assembled DNA w/o Ddx4 (Supplementary Fig. S8B, right). To evaluate the assembly fidelity, plasmid DNA from colonies was subjected to sequencing. Among clones derived from droplet-enhanced assembly, 41.7% (at 5 nM) and 33.3% (at 0.5 nM) had entirely correct sequences (Fig. 4B). 29.2% (5 nM) and 35.6% (0.5 nM) of clones exhibited sporadic 1–3 nucleotide errors including substitution or deletion, originating from oligo synthesis as discussed below. A further 20%–30% of clones contained long deletions between correctly assembled regions, possibly resulting from misassembly between truncated oligos contaminating in the original oligo mixture. The remaining 8.3% (5 nM) and 11.6% (0.5 nM) were unreadable reads. Notably, all clones obtained from assembled DNA w/o droplets displayed only unreadable sequence results.

To assess the impact of oligo synthesis errors, we analyzed the individual oligos by NGS without assembly reaction (Fig. 4C and Supplementary Fig. S9). The error rate was 0.151% per base (Supplementary Fig. S9). Assuming ligation occurs only when the 20-nt overlap regions are perfectly matched, the calculated probability of generating a completely error-free assembly was 40.94%, which aligns closely with the experimentally observed frequency of correct clones (41.7% and 33.3%). We also analyzed the types of synthesis errors of the individual oligo—deletions, substitutions, and insertions—that were found at the frequencies of 71.4%, 27.6%, and 1.3%, respectively (Fig. 4C). These distributions were consistent with those observed in the droplet-enhanced assembly: 77.4%, 22.6%, and 0.00%. These findings confirm that the sporadic sequence errors observed in the assembled clones primarily originated from oligo synthesis. Within the screening capacity of this pipeline, we have successfully validated the assembly and expression of DNA fragments up to 900 bp.

### Digital cell-free gene expression of assembled DNA

**Figure 5.**
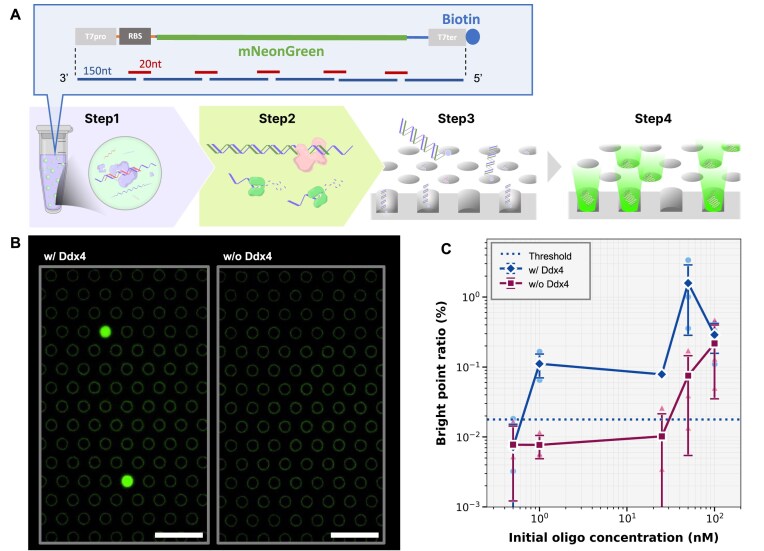
Digital Cell-free gene expression from assembled DNA (**A**) The schematic image of cell-free protein prototyping integrating cell-free digital gene expression and droplet-enhanced oligo assembly. (**B**) Fluorescence microscope image of femto-liter reactors expressing mNeonGreen protein from droplet-enhanced assembly (left) and assembly w/o droplet (right). Initial oligo concentration was 1 nM. (**C**) Fraction of reactors expressing mNeonGreen protein with bright fluorescence signal (*N* = 3). The threshold line is defined as mean +3 s.d. of the pseudo-positive signal (the bright reactor fraction in the absence of DNA); 1.79 × 10^−2^.

Here, we investigated the gene expression capability of droplet-enhanced oligo assembly under cell-free conditions to explore its potential for rapid protein prototyping. For this purpose, we designed an oligo set incorporating a T7 promoter and ribosome binding site (RBS) upstream of the mNeonGreen gene, and a T7 terminator downstream. The expected total length of the assembled DNA was 858 bp (Fig. 5A). The 5′-terminal of the oligo that corresponds to the 5′ end position of the full DNA construct, was biotinylated for surface immobilization on the bottom of femtoliter reactors, as described below.

Two major challenges hinder direct gene expression from DNA of droplet-enhanced oligo assembly. First, the amount of assembled DNA is extremely low: qPCR analysis showed that assembly from a 5.0 nM oligo mixture yielded only 1.2 fM of full-length DNA (Supplementary Fig. S7C), which is not sufficient for conventional bulk-scale cell-free expression at all. Second, the reaction mixture contains over 10^6^-fold excess of unassembled or partially assembled oligos, which significantly interfere with gene expression (Supplementary Fig. S10).

To address these challenges, we employed a digital gene expression method using a FRAD [26]. The FRAD generates ~10^5^–10^6^ micron-sized water-in-oil droplets, each with a volume of ~50 fL that allows the micro-compartmentalization of template DNA molecule as well as expressed proteins, enabling sensitive detection of gene expression activity from single template DNA molecules. Furthermore, we introduced two additional modifications in the protocol for the purpose to mitigate the interfering effect of incomplete assemblies or excess oligos. The first modification involved a buffer wash after DNA immobilization on the device. The 5′-biotinylated oligo enabled selective attachment of assembled DNA having biotinylated 5′ end to the streptavidin-coated bottom of the micro reactors. A subsequent buffer wash removed DNA fragments lacking 5′ biotin. The second modification was Exonuclease I treatment following strand extension by Taq polymerase. The primer for extension was designed to synthesize the plus strand from the 3′ end of the full DNA construct. As a result, incomplete assemblies lacking the 3′-terminal oligos remained single-stranded and were selectively digested by Exonuclease I. The effectiveness of Exonuclease I treatment was confirmed experimentally (Supplementary Fig. S11).

The overall assay workflow is illustrated in Fig. 5A. (Step 1) Oligos were assembled in droplets. (Step 2) The assembled DNA products were converted to double-stranded structure by Taq polymerase and treated with Exonuclease I. (Step 3) The reaction mixture was loaded into the FRAD, and biotinylated DNA molecules were immobilized at the single-molecule level. Nonimmobilized fragments were removed by buffer wash. (Step 4) Cell-free expression mixture was added, and the device was incubated at 37°C to initiate digital gene expression.

Figure 5B shows fluorescence images of the digital gene expression activity in the femtoliter microreactors with DNA of droplet-enhanced assembly DNA or w/o Ddx4. At 1 nM oligo input with Ddx4 droplets, several reactors exhibited fluorescence among a large number of dark reactors, demonstrating the digital nature of gene expression. In contrast, no fluorescent reactors were detected from the assembly w/o Ddx4 except for the pseudo-positive signal that occurred at the frequency of 1.79 × 10^−2^%.

For quantitative comparison, we defined the threshold oligo concentration as the minimum concentration that yields a frequency of fluorescent reactors exceeding the pseudo-positive level. This threshold was between 0.5 and 1 nM for droplet-enhanced assembly (Fig. 5B and C), corresponding to several femtomoles—within the yield of commercially available oligo pools. In contrast, the threshold for assembly w/o droplet was between 25 and 50 nM (Fig. 5C), indicating a 25–100-fold enhancement in digital gene expression by droplet-enhanced assembly.

To confirm that the enhancement is attributable not to direct interaction of oligo and Ddx4 protein but to the condensation effect by Ddx4 droplet, we performed digital gene expression assays using assembled DNA prepared with varying concentrations of Ddx4 (Supplementary Fig. S12). The enhancement was observed only at Ddx4 concentrations above the critical threshold for phase separation, confirming that the formation of Ddx4-based droplets is key to enhancing the DNA assembly process. The results also indicated that increasing the Ddx4 concentration beyond the threshold required for droplet formation produced only marginal effects.

### Parallel expression of multiple proteins from oligos

To demonstrate the versatility of the droplet-enhanced oligo assembly integrated with cell-free digital gene expression, we tested oligo assembly and digital gene expression of mScarlet, starting from 1 nM oligo mix (Supplementary Fig. S13). In the presence of phase-separated droplets, fluorescent reactors (“bright points”) were observed at frequencies exceeding the detection threshold. In contrast, no bright points were detected without droplets.

**Figure 6.**
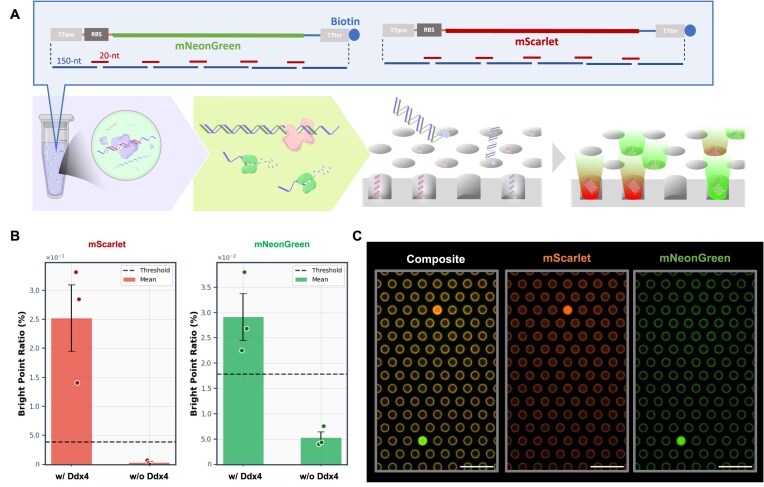
Dual gene expression from assembled DNA. (**A**) The schematics of dual gene expression from assembled DNA. (**B**) Fractions of positive reactor found in mScarlet channel (red) or in mNeonGreen channel (green) (*N* = 3). The initial oligo concentrations were 1 nM. Dotted lines represent the thresholds defined as mean +3 s.d. of pseudo-positive signal (the bright reactor fraction in the absence of DNA); the values are 3.91 × 10^−2^ for the mScarlet channel and 1.79 × 10^−2^ for the mNeonGreen channel. (**C**) Fluorescence microscope images of femto-liter reactors expressing mScarlet and mNeonGreen protein synthesized via droplet-enhanced assembly.

We further validated the ability of the system for parallel gene expression. Oligos encoding both mNeonGreen and mScarlet were co-assembled in a single reaction and subjected to simultaneous digital gene expression assay (Fig. 6A). At an input concentration of 1 nM, both proteins were successfully expressed and detected as distinct fluorescent signals (Fig. 6B and C), ensuring the applicability for multiple gene expression. Without Ddx4 droplets, no fluorescent reactors were observed at the same concentration (Fig. 6B).

## Discussion

The present study showed that the integrated oligo-to-protein expression system enables rapid protein expression from very low quantity of synthetic oligos (as low as 0.5–1 nM), completing the entire workflow in half a day. The timeline includes 1 h for oligo assembly, 1 h for post-assembly processing, 1 h for FRAD immobilization and washing, and 6–8 h for cell-free protein expression. Time-course experiments (Supplementary Fig. S14) showed that while average fluorescence intensity peaked at 8 h, the percentage of positive reactors plateaued at 6 h. Thus, depending on the application—quantification or detection—protein expression can be assessed in 6–8 h. This represents a significant reduction from conventional workflows, which require at least a few days including amplification, assembly, cloning, transformation, and expression [7, 12]. Importantly, we also demonstrated the parallel expression of two proteins from distinct oligo sets in a single reaction, underscoring the system’s potential for scalable, multiplexed protein prototyping.

Notwithstanding these advantages, interference from unreacted or partially assembled oligos remains a limiting factor. We observed that protein expression efficiency decreased as the total oligo concentration increased (Fig. 5). Comparative experiments with or without excess unassembled oligos (Supplementary Fig. S15) indicated that residual oligos likely impair either Taq polymerase-mediated strand extension and/or streptavidin-based immobilization. This interference likely stems from primer competition or streptavidin saturation by free biotin. Since both mechanisms are saturable, the effect becomes more pronounced at higher oligo concentrations. In addition, regarding oligo assembly, sequencing suggested misassembly between truncated oligos contaminating the original oligo mixture (Fig. 4). Although no large-scale rearrangements were detected, the probability of such misassembly may increase with the complexity of the oligos, reducing the fraction of correctly assembled sequences. This phenomenon was particularly evident in parallel expression assays using mScarlet and mNeonGreen at a 1 nM input concentration, where expression efficiency was reduced compared to single-protein expression (Supplementary Fig. S16).

To achieve further parallelization and scalability, relying solely on bulk one-pot multiplexing is likely insufficient due to the above-mentioned inherent constraints such as primer competition, and misassembly. To overcome these limitations, we envision a spatially defined strategy—termed FRAD-based protein prototyping—in which predefined sets of oligos are captured and assembled within individually addressable microcompartments. Conceptually, this approach parallels droplet-based partitioning methods like DropSynth but utilizes Ddx4 droplets within femtoliter reactors to mediate *in situ* assembly. By localizing specific oligo sets to isolated positions, this spatial confinement prevents cross-reactivity, thereby enabling high-throughput and scalable analysis.

Importantly, such spatial confinement also enables preservation of the gene–function link. When predefined oligo sets are assembled within specific microreactors, the physical location itself serves as an index connecting genotype and phenotype. Alternatively, even if defined oligo sets are captured randomly within compartments, sequence–function linkage could be established through post-expression sequence recovery. For example, our previously reported single-molecule DNA recovery and analysis technique using microneedles [21] provides a feasible route to retrieve genetic information from individual microreactors after functional screening.

To examine the feasibility of these concepts, we experimentally demonstrated that IDP droplets can form within individual FRAD reactors and enrich oligo DNA in confined femtoliter environments (Supplementary Fig. S17). The results confirmed the presence of spherical condensates within the reactors enriching the oligos, whereas no such particles were observed in the control experiment lacking Ddx4. Theoretically, assuming an initial 1 nM bulk concentration and a two- to three-fold up-concentration via spatial confinement, the effective oligo concentration within the reactor reaches ~2–3 nM. This falls well within our optimal assembly range of 1–25 nM. While this experiment successfully demonstrates up-concentration, the actual oligo assembly reaction within these femtoliter droplets has not yet been experimentally achieved. Nevertheless, it validates the physical compatibility of Ddx4-mediated enrichment with spatially compartmentalized assembly, providing a plausible path for future development.

To extend the utility of FRAD-based protein prototyping to large-scale scenarios involving oligo pool amplification, ensuring efficient assembly under low-concentration conditions is essential. In parallel synthesis methods like DropSynth, the total PCR yield is divided among thousands of variants, resulting in individual oligo concentrations in the nM range. Furthermore, stochastic amplification bias can further lower specific concentrations to 0.01–0.1 nM during the assembly reaction. To address this practical challenge, we evaluated the Ddx4 droplet method for Golden Gate Assembly as a post-PCR oligo assembly strategy (Supplementary Fig. S18A). Our qPCR analysis revealed that Ddx4-mediated droplet formation significantly improves assembly efficiency in these low-concentration regimes (0.01–1 nM). Specifically, at 0.1 nM, the efficiency increased 22.4-fold (1.14% with Ddx4 versus 0.05% without, Supplementary Fig. S18). These findings suggest that Ddx4 is a potent solution for overcoming the concentration barriers inherent in post-PCR oligo assembly.

The physical environment within Ddx4 droplets presents a complex balance between molecular enrichment and reaction kinetics. Quantitative qPCR analysis in Fig. 3 revealed that the assembly efficiency from 0.5 fmol oligos with Ddx4 was several-fold lower than that from 5 fmol oligos in a bulk solution, despite a 10- to 100-fold increase in local oligo concentration within the droplets. This discrepancy suggests that while Ddx4-based droplets effectively sequester reactants, they simultaneously attenuate the intrinsic assembly efficiency per molecule. At higher initial oligo concentrations, the use of Ddx4-based droplets becomes counterproductive, resulting in a net assembly yield lower than that of droplet-free controls (Fig. 3). We hypothesize that as the local concentration within the droplets reaches a saturation point for assembly activity, the inhibitory effects of the microenvironment negate and eventually outweigh the kinetic benefits of molecular enrichment. This reduction in efficiency may stem from the restricted molecular diffusion within the condensed phase or destabilization of double-stranded intermediates.

The stability of nucleic acid structures within these droplets is also highly sensitive to the physicochemical environment, particularly salt concentration. While our optimized conditions prevented significant DNA destabilization by Ddx4 droplets, previous studies have reported that the droplets can destabilize or exclude double-stranded structure [23]. In contrast to the prior work using 150 mM NaCl, the present work employed lower NaCl concentrations (<27 mM), under which we observed no exclusion or dissociation of dsDNA (Fig. 1). However, at elevated salt (81 mM), dsDNA accumulation in droplets was reduced, and distinct localization patterns emerged (Supplementary Fig. S19I-iii and Supplementary Table S1), consistent with partial duplex destabilization. These results suggest that NaCl concentration is a key determinant of duplex stability in Ddx4 droplets. One hypothesis is that NaCl weakens the electrostatic interactions between Ddx4 and DNA through a shielding effect, and that a reduction in this shielding effect may promote DNA concentration. Furthermore, time-course measurements revealed slow duplex dissociation under droplet conditions (Supplementary Fig. S5). While Ddx4 performed adequately under our current conditions, these findings highlight the potential need for future refinement of Ddx4 properties or finding of more appropriate droplet systems to ensure compatibility with diverse nucleic acid structures, particularly for applications requiring stable duplex formation.

In summary, we have developed an amplification-free, cell-free platform that enables rapid protein expression from low-input synthetic oligos within half a day by integrating Ddx4-mediated assembly with digital gene expression in femtoliter reactors. By bypassing post-assembly PCR, plasmid construction, and cellular cloning, this workflow significantly reduces hands-on time and complexity compared to established methods. Although its current capacity for parallelization remains more restricted than mature approaches, this study establishes a critical technological foundation for future FRAD-based protein prototyping. Ultimately, by scaling overall throughput while preserving the hallmark simplicity and rapid turnaround of this workflow, this platform can evolve into a versatile tool for high-capacity protein engineering.

## Supplementary Material

gkag431_Supplemental_Files

## Data Availability

The data underlying this article are available in the article and in its online supplementary material. Codes for data analysis are provided in supplementary files with this paper.
