# Supplementary material for: Intrinsically disordered protein droplet-enhanced oligonucleotide assembly enables rapid oligonucleotide-to-protein expression
Source: Nucleic Acids Res. 2026 May 4;54(8):gkag431. doi: 10.1093/nar/gkag431 (PMC13136894; doi:10.1093/nar/gkag431)
Supplement: gkag431_Supplemental_Files [file gkag431_supplemental_files.zip › Supplementary_Information.pdf]

Supplementary Information

Intrinsically disordered protein droplet-enhanced oligonucleotide assembly enables rapid oligonucleotide-to-protein expression

Taiji Ueno[1], Yoshihiro Minagawa[1], Yasushi Okada[2-5], Hiroyuki Noji[1,6] *

[1]Department of Applied Chemistry, Graduate School of Engineering, The University of Tokyo, Tokyo, Japan.

[2]Department of Physics, and Universal Biology Institute (UBI), Graduate School of Science, The University of Tokyo, Hongo, Tokyo, Japan

[3]Laboratory for Cell Polarity Regulation, RIKEN Center for Biosystems Dynamics Research, Kobe, Hyogo

[4]Department of Cell Biology, Graduate School of Medicine, The University of Tokyo, Hongo, Tokyo

[5]International Research Center for Neurointelligence (WPI-IRCN), The University of Tokyo, Hongo, Tokyo

[6]Research Institute of Planetary Health (RIPH), The University of Tokyo, Tokyo, Japan.

*Corresponding authors: hnoji@g.ecc.u-tokyo.ac.jp

This pdf includes:

Supplementary Methods
Supplementary Figures: Figure S1-20
Supplementary Tables: Table S1-4

Supplementary Materials and Methods

Derivation of the Condensate-Specific Correction Factor (γ_{in})

The FRET correction factor, γ, represents the ratio of the effective fluorescence detected from the acceptor (sensitized emission) to the fluorescence loss of the donor (quenching):

$$\gamma = \frac{\phi_A \cdot \eta_A}{\phi_D \cdot \eta_D}$$

where ϕ is the quantum yield and η is the detection efficiency of the instrument (including laser power, optical transmission, and detector sensitivity). In this study, the η was assumed to be identical as for FAM (Donor) and Cy5 (Acceptor), and thus, $\gamma \approx \frac{\phi_A}{\phi_D}$. The γ value outside droplets was therefore calculated from literature quantum yields as:

$$\gamma_{out} = \frac{\phi_{A\_out}}{\phi_{D\_out}} = \frac{0.20}{0.93} \approx 0.215 \ (\phi_{D\_out} = 0.93,\ \phi_{A\_out} = 0.20 \ (1))$$

Fluorescence lifetime measurements confirmed a significant decrease in donor quantum yield inside the condensates: Donor Lifetimes (τ): $\tau_{bulk} = 3.92\,\mathrm{ns}$, $\tau_{droplt} = 1.69\,\mathrm{ns}$ (Supplementary Figure S2B). Quantum yield shift factor (F_D): Assuming $\phi \propto \tau$, the donor quantum yield decreased by a factor of

$$\frac{\phi_{D\_in}}{\phi_{D\_out}} = \frac{1.69}{3.92} \approx 0.431$$

Assuming that the distribution ratios of the dyes are identical, the ratio of the obtained Cy5 intensity to the FAM intensity (FAM/Cy5), together with the FLIM results, indicates

$$\frac{\phi_{A\_in}}{\phi_{A\_out}} = \frac{0.431 \cdot 511}{180} \approx 1.22$$

(Supplementary Figure S2D). the FRET correction factor inside the droplets was estimated as

$$\gamma_{in} \approx \frac{\phi_{A_{in}}}{\phi_{D_{in}}} = \frac{1.22}{0.431} \times 0.215 = 0.61$$

The calculated $\gamma_{in} \approx 0.61$ indicates although donor quenching is enhanced inside the condensates, the relative effective emission from the acceptor compensates for this effect, making this value the most appropriate for radiometric FRET analysis in this environment.

The FRET efficiency inside the droplets was then calculated as

$$FRET\ efficiency_{\ in} \approx \frac{I_{A\_in}}{I_{A\_in} + 0.61\ \cdot I_{D\_in}}$$

Next Generation Sequencing of Synthesized Oligos

DNA libraries were prepared using the NEBNext Ultra II DNA Library Prep Kit for Illumina. Adapter-ligated DNA (15 μL) was mixed with NEBNext Ultra II Q5 Master Mix (25 μL), Index Primer/i7 Primer (5 μL), and Universal PCR Primer/i5 Primer (5 μL) in 50 μL reactions. PCR amplification was performed using the following cycling conditions: 98°C for 30 s, followed by 3 cycles of 98°C for 10 s and 65°C for 75 s, with a final extension at 65°C for 5 min.

PCR products were purified using SPRIselect (45 μL, Beckman Coulter) with 5 min incubation at room temperature. Beads were collected on a magnetic stand for 5 min, washed twice with 80% ethanol (200 μL each), and air-dried for 5 min. DNA was eluted in 33 μL of 10 mM Tris-HCl, and 30 μL of supernatant was collected after magnetic separation.

DNA concentrations were measured using the QuantiFluor ONE dsDNA System (Promega). Standards and samples were prepared by mixing 1-2 μL DNA with 200 μL QuantiFluor ONE dsDNA Dye, incubated for 5 min at room temperature protected from light, and measured using a Quantus Fluorometer. Individual samples were pooled at equal molar concentrations (0.20 ng/μL each) and diluted to 200 pM for sequencing.

Pooled libraries were sequenced on an iSeq 100 system (Illumina). The sequencing cartridge was equilibrated to room temperature, mixed by inversion (5 times), and tapped on the bench (5 times). Sample loading wells were punctured with the provided needle, and 20 μL of 200 pM pooled DNA was loaded. Sequencing was performed according to manufacturer's protocols with custom index sequences and adapter trimming settings.

Raw sequencing data were processed using custom Jupyter Notebook scripts (NGS_data_analysis.ipynb provided at Supplementary Source Codes). Sequences were filtered for minimum length (100 nt) and quality score (Q20 per base). PairwiseAligner was used for reference mapping with parameters: match score +1, mismatch penalty -2, gap penalty -2.5. Mutations, deletions, and insertions were counted in the region from 3 nucleotides from each terminus. Error rates were calculated as events per nucleotide, and mutation patterns were visualized using matplotlib. Sequences with consecutive deletions ≥3 nucleotides were analyzed separately to distinguish synthesis errors from other artifacts.

Image Analysis and Fluorescence Quantification

Fluorescence images of expressed proteins within femtoliter reactors were acquired using an epifluorescence microscope equipped with appropriate filter sets for mNeonGreen (mNG) and mScarlet (mSc) detection. Images were captured using a Nikon Apo 60× 1.40 NA oil immersion objective (λS ∞/0.17 DIC N2). Imaging parameters were as follows: AlexaFluor647 (excitation: 630 nm at 20% intensity; filter set - emission: 670 nm peak, excitation: 625-650 nm, dichroic mirror: 625 nm cutoff; exposure time: 50 ms), mNeonGreen (λ_{ex}: 475 nm at 100% intensity; filter set – λ_{em}: 510-560 nm, excitation: 460-500 nm, dichroic mirror: 505 nm cutoff; exposure time: 200 ms), and mScarlet (λ_{ex}: 575 nm at 100% intensity; filter set - emission: 590-650 nm, λ_{em}: 528-553 nm, dichroic mirror: 565 nm cutoff; exposure time: 200 ms). For each sample, 288 positions per channel were acquired and saved in multi-channel format.

Raw images were processed using ImageJ software with custom macros (Fiji_image_modification.ijm and Fiji_ROI_measurement.ijm provided at Supplementary Source Codes). Dark current and flat-field corrections were

applied to all images. Automated quality control was implemented using a custom ImageJ macro to ensure data consistency. For each time point, statistical parameters including mean intensity and standard deviation were calculated within a central region of interest (ROI). Images were automatically excluded based on the following criteria: (1) standard deviation values exceeding 1.1-fold the mean standard deviation across all images, or (2) mean intensity values deviating by more than one standard deviation from the overall mean intensity. Quality-controlled frames were compiled into substacks for subsequent analysis. Automated particle detection was performed on the AlexaFluor647 channel using ImageJ's "Analyze Particles" function. Detection parameters were optimized to identify circular objects with areas of 10-40 pixels and circularity values of 0.80-1.00. Initial ROIs were refined using a custom macro that calculated centroid coordinates for each detected particle and generated standardized circular ROIs. Two ROI sets were created for each detected particle: small ROIs (8 × 8 pixels) centered on particle centroids for precise fluorescence measurements, and large ROIs (30 × 30 pixels) for background assessment and validation purposes. Fluorescence intensities for mNeonGreen and mScarlet channels were measured separately using ImageJ's ROI Manager. Measurements included mean intensity, standard deviation, area, and spatial coordinates (X, Y) with corresponding slice numbers for time-lapse sequences. Data were exported as comma-separated value (CSV) files, with separate files generated for each fluorescent protein and experimental condition. Fluorescence data analysis was conducted using Jupyter Notebook scripts (FRAD_data_analysis.ipynb provided at Supplementary Source Codes) incorporating pandas, NumPy, and matplotlib libraries. Intensity distributions were analyzed through histogram analysis with 1000 bins. Gaussian mixture models were fitted to determine intensity thresholds for positive signal detection. For standard deviation analysis, single Gaussian distributions were fitted with detection thresholds set at the mean plus three standard deviations. For mean intensity analysis of mNeonGreen, single Gaussian fits were applied with thresholds established at the mean plus five standard deviations. A multi-step filtering protocol was implemented to eliminate false-positive detections. First, particles were filtered based on mean intensity and coefficient of variation

thresholds derived from Gaussian fits. Second, particles detected within small ROIs (8 × 8 pixels) were validated against corresponding large ROI measurements (30 × 30 pixels); particles were retained only when small ROI intensities exceeded large ROI intensities. Third, proximity-based filtering removed particles within 12 pixels of each other within the same time frame to eliminate clustering artifacts. Final particle counts and average fluorescence intensities were calculated for each experimental condition.

Golden Gate Assembly and qPCR measurements

Golden Gate Assembly was performed in 20 μL reactions containing $Ddx4^{N1}$ (75 μM), oligo mixture (10nM -1 pM final concentration of each oligo), 1× T4 DNA Ligase Buffer contained in NEBridge® Golden Gate Assembly Kit (BsaI-HF® v2) and 1μL of NEB Golden Gate Assembly Mix. The oligo set used was Oligo Assembly System 5 in Table S2. Reactions were incubated at 37°C for 1 h, followed by enzyme inactivation at 60°C for 5 min. Control reactions without $Ddx4^{N1}$ were performed under identical conditions.

Oligo assembly efficiency was quantified by SYBR qPCR using specific primers and hydrolysis probes designed to amplify assembled products (sequences in Supplementary Table S2). qPCR was performed in 20 μL reactions containing forward and reverse primers (each at 0.1 μM final concentration), SYBR Green I (x1 final concentration) and 1× KOD one master mix. Two microliters of the assembled products were added to each 20 μL qPCR reaction as template. If the used initial concentration was over 1nM, the assembled products were diluted 1:10 in nuclease-free water. Reactions were run on an CFX Opus 96 (Bio-Rad) with the following cycling conditions: [initial denaturation at 95 °C for 2 min, followed by] 60 cycles of 98 °C for 10 s and 68 °C for 5 s. Fluorescence was recorded during the annealing/extension step. For quantification of assembly efficiency, three independent assembly reactions were analyzed (n = 3 biological replicates), and each sample was measured once by qPCR (no technical replicates). Ct values were determined using the automatic threshold setting of the instrument software. Standard curves for the ligation junction assays were described by DNA amount = 6.16×10^7 × exp

(−0.67 × Ct), corresponding to slopes of −3.44 in Ct versus $\log_{10}$ (DNA concentration) plots and amplification efficiencies of approximately 95%, respectively (R^2 = 0.997; Supplementary Figure S18).

Supplementary Figures and Tables

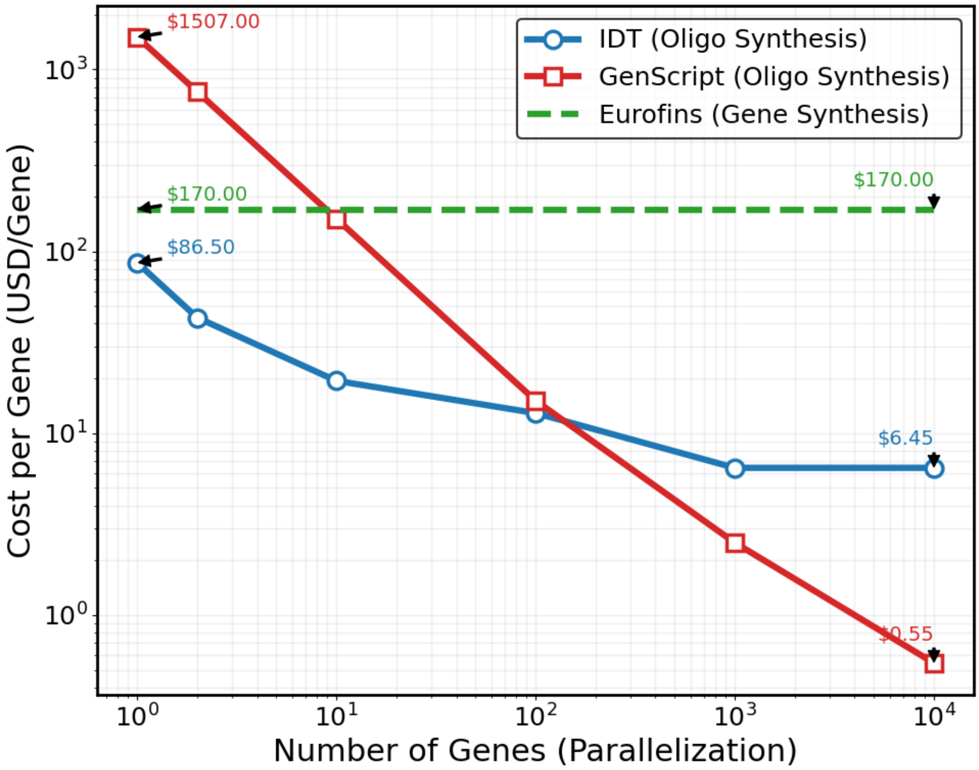

Supplementary Figure S1: Economic trade-offs between starting materials and pre-assembled gene fragments. Unit costs for gene synthesis are compared across three providers. Pricing for IDT and GenScript reflects the cost of oligo pools used as the starting material for de novo gene construction; these figures do not include the labor or reagent costs associated with downstream assembly. Eurofins pricing denotes the direct cost of sequence-verified gene fragments. Note that while GenScript offers superior scalability in oligo pool pricing—dropping below $1 USD per gene at the 10,000-gene scale—this requires a robust internal assembly pipeline. Eurofins provides a consistent price point for ready-to-use fragments, independent of the parallelization scale. The graph was created based on Supplementary Table S4.

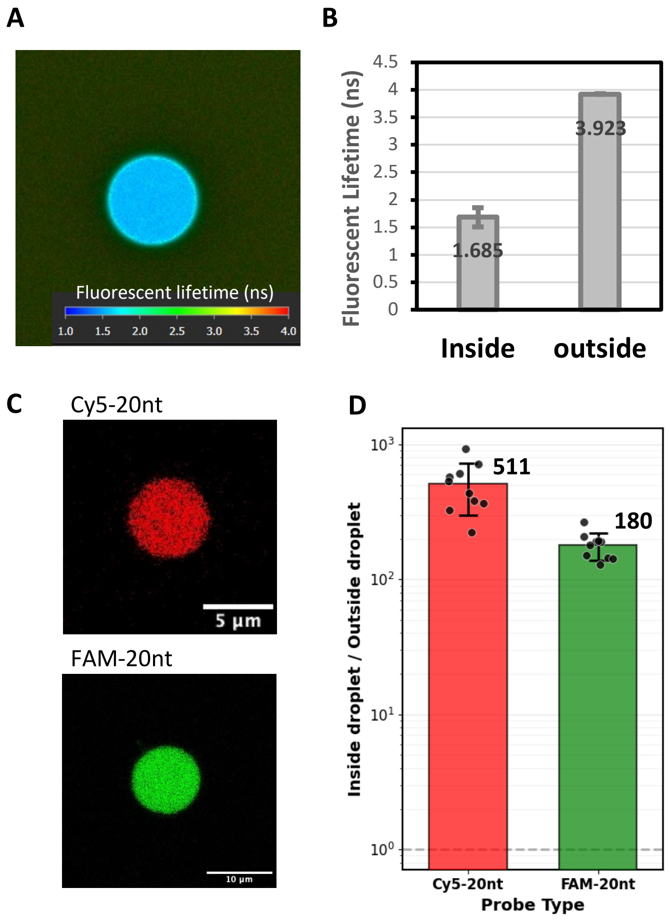

Supplementary Figure S2: Experiment for partition coefficient correction. **(A)** Image shows the result of FLIM. **(B)** The results show the Fluorescent lifetime inside or outside of the droplets. N=3. The correction value (inside/outside) is 2.33 and this value was multiplied by the ratio of internal to external measurements obtained using FAM or fluorescein labeled DNA to derive the partition coefficient. **(C)** Comparison between labelling with Cy5 and labelling with FAM **(D)** Comparison of the ratio of internal to external fluorescence intensities when using FAM and Cy5. Here, assuming the two actual distribution ratios are identical, the ratio of the obtained Cy5 value to the FAM value (FAM/Cy5) and the previous FLIM results indicate that when measuring the distribution ratio of substances labelled with Cy5 or its approximate equivalent Alexa647, a factor of 0.82 (= 2.33 × 180 / 511) should be applied.

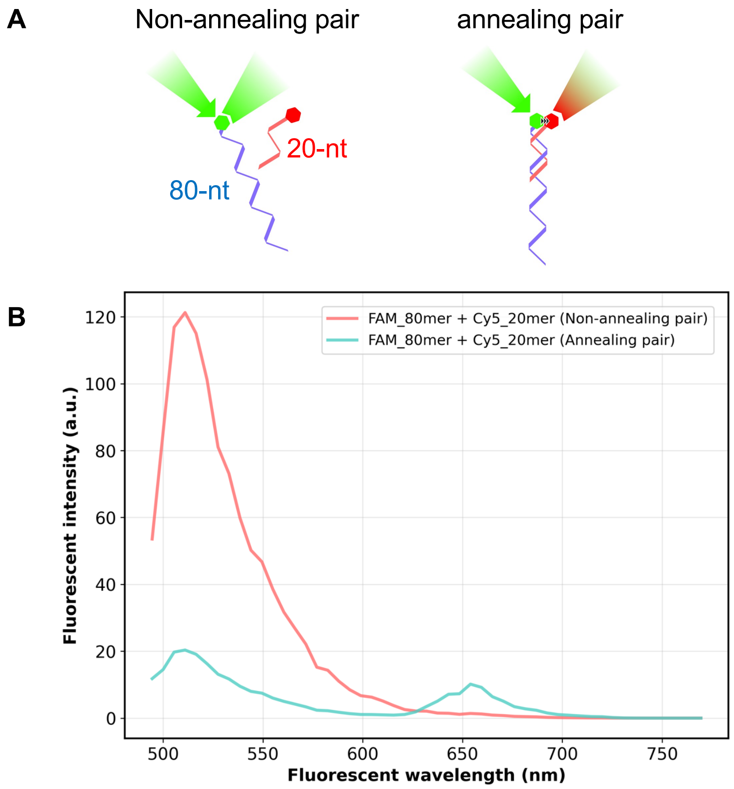

Supplementary Figure S3: Confirmation of FRET probe (**A**) Schematic illustration of complementary (annealing) and non-complementary (non-annealing) FRET probe pairs. The annealing pair consists of oligos designed to form stable base-paired duplexes, bringing donor and acceptor fluorophores into proximity for efficient FRET. The non-annealing pair serves as a negative control with no sequence complementarity. (**B**) Fluorescence emission spectra of both FRET probe pairs measured in storage buffer without Ddx4-induced phase separation. Excitation wavelength: 495 nm. Spectra were acquired at 5.5 nm intervals across 400-784 nm using a Leica spectroscopic system. Key spectral features: fluorescence at 510 nm represents direct FAM emission (F_D, donor fluorescence), while fluorescence at 650 nm represents FRET-mediated emission (F_A, acceptor fluorescence upon donor excitation).

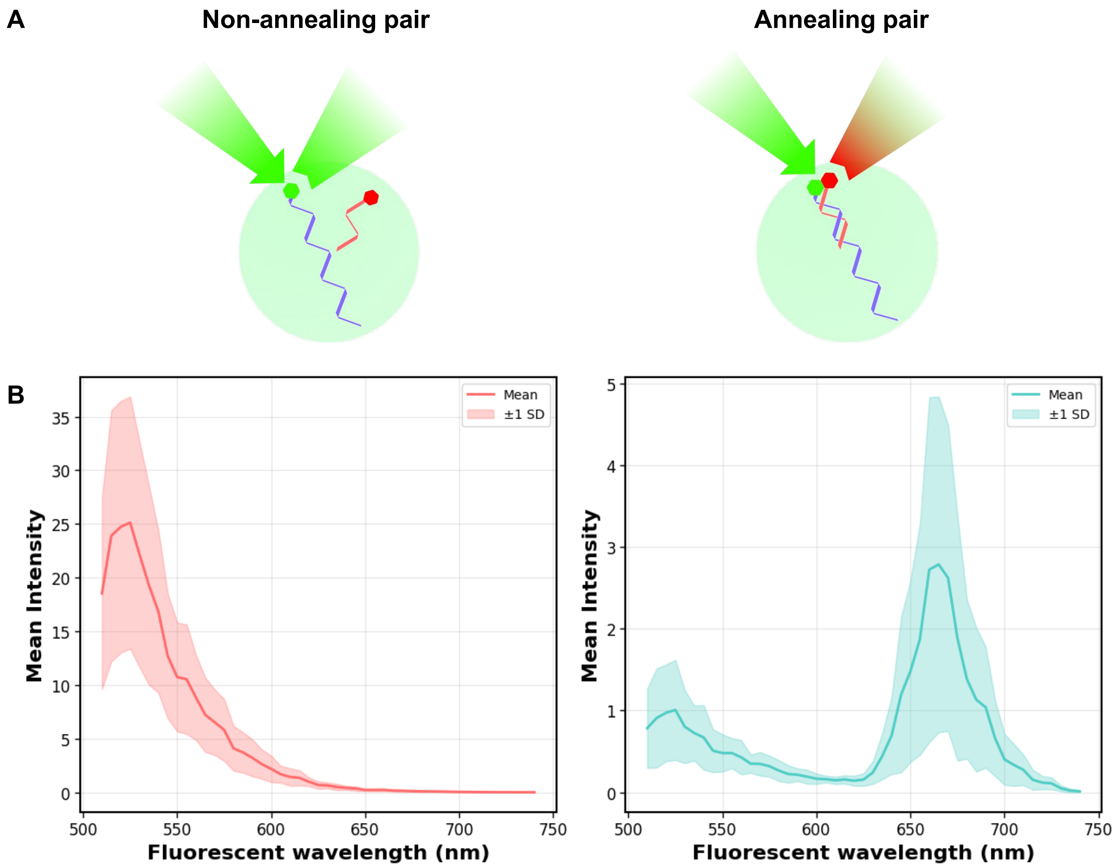

Supplementary Figure S4: Spectra of FRET probe inside the phase separated droplets (**A**) Schematic illustration of FRET probe distribution within condensate droplets. Left panel: non-annealing (non-complementary) probe pair showing random distribution with minimal FRET interaction. Right panel: annealing (complementary) probe pair demonstrating enhanced proximity and FRET efficiency due to base-pairing interactions within the condensed phase. (**B**) Fluorescence emission spectra of FRET probe pairs measured within condensate droplets. Excitation wavelength: 495 nm. Spectra were acquired at 5.5 nm intervals across 400-784 nm. Key spectral features: fluorescence at 510 nm represents direct FAM emission (F_D, donor fluorescence), while fluorescence at 650 nm represents FRET-mediated emission (F_A, acceptor fluorescence upon donor excitation). Left panel: spectral data from non-annealing probe pairs. Right panel: spectral data from annealing probe pairs. Each condition represents measurements from 10 individual droplets.

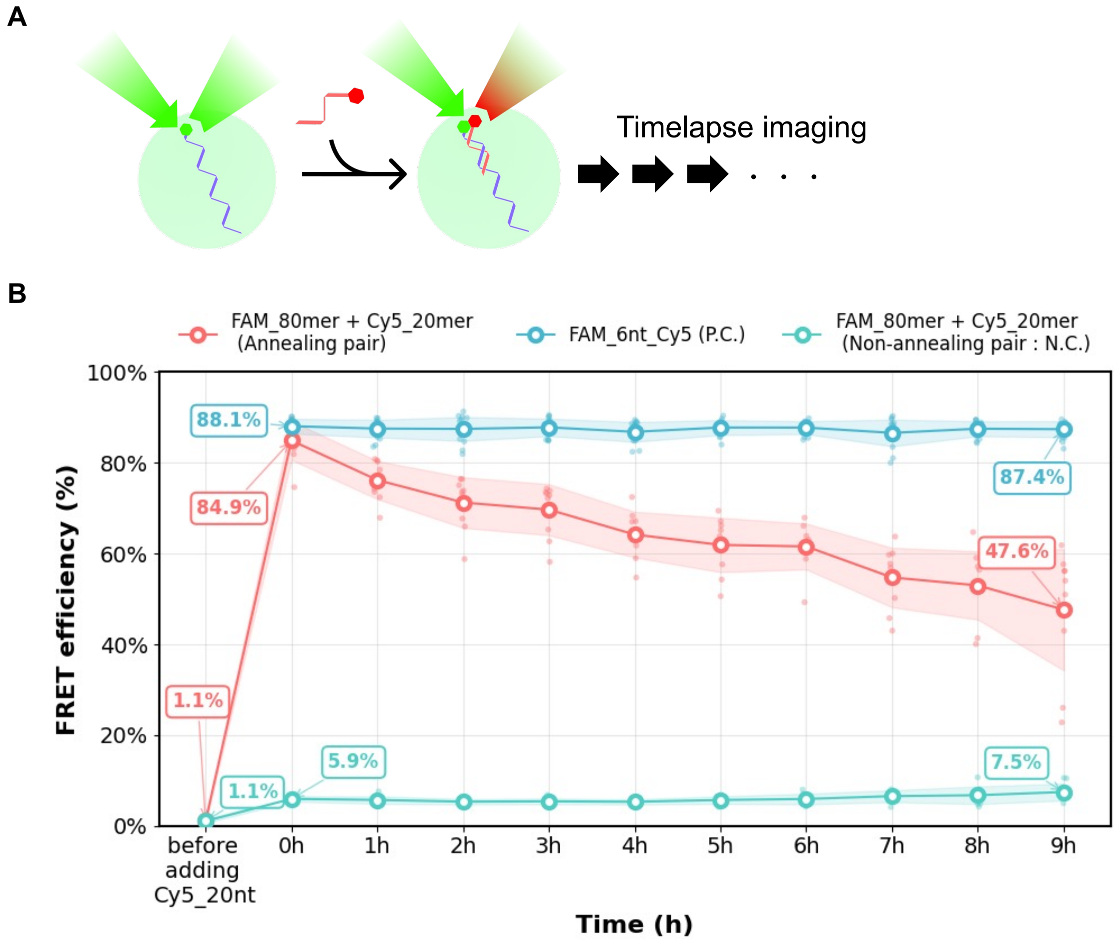

Supplementary Figure S5: Timelapse analysis of FRET efficiency inside the droplets (**A**) Schematic image of the experiment which demonstrate the annealing inside the droplet using the FRET probe. In this experiment, timelapse imaging was additionally performed after the same experiment shown in Figure 2 (**B**) The results of the experiments showing their FRET efficiency of timelapse. How was the FRET efficiency calculation descripted in the Methods section. Annealing pair means the result using the Cy5 attached 20-nt ssDNA which can anneal on the FAM attached 80nt ssDNA. Non-annealing pair means the result using the Cy5 attached 20-nt ssDNA which cannot anneal on the 80nt ssDNA. FAM_6nt_Cy5 means the result using 6 nt ssDNA with FAM at 3' end and Cy5 at 5' end, in which the distance between the Cy5 and FAM is almost same as the FRET probe. N=10 for each condition. The FRET efficiency was calculated as FA / (FA + γFD). The procedure for calculating γ is described in the Supplementary Materials and Methods.

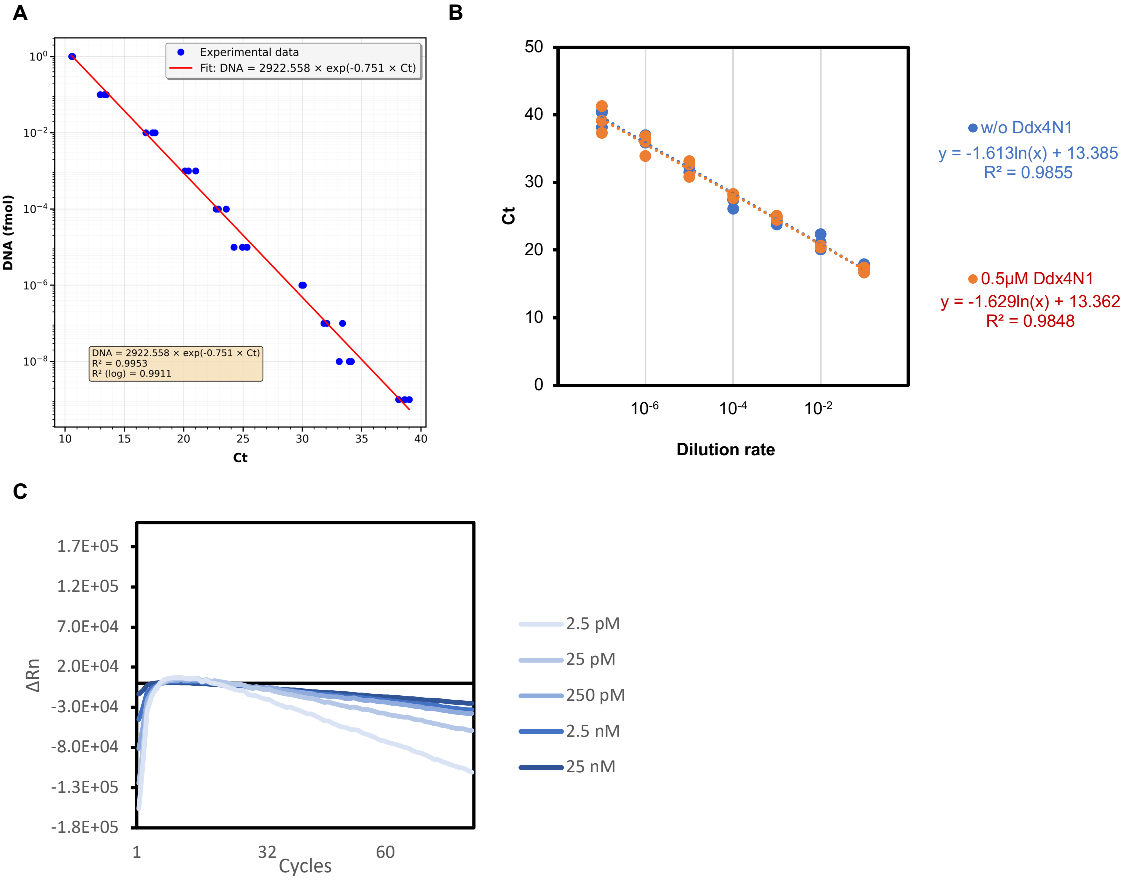

Supplementary Figure S6: Confirmation of quantifiability of qPCR in designed oligo assembly (**A**) Calibration curve for qPCR. DNA with all oligos assembled and duplex DNA encoding the same sequence were diluted in steps to obtain their respective Ct values. n=3 for each concentration. (**B**) Results of qPCR on samples in which oligo assembly was performed without phase separation at an initial oligo concentration of 250 nM, and serial 10-fold dilutions were performed. At this time, two patterns were created and experimented with and without Ddx4 added during qPCR. The results showed that the plot of dilution factor vs. Ct value was on a straight line, and the linearity did not change with the presence or absence of Ddx4s. (**C**) Results of qPCR on unlinked oligos. n=3 for 25 nM, 2.5 nM, 250 pM, 25 pM and 2.5 pM, respectively. No increase in signal was observed.

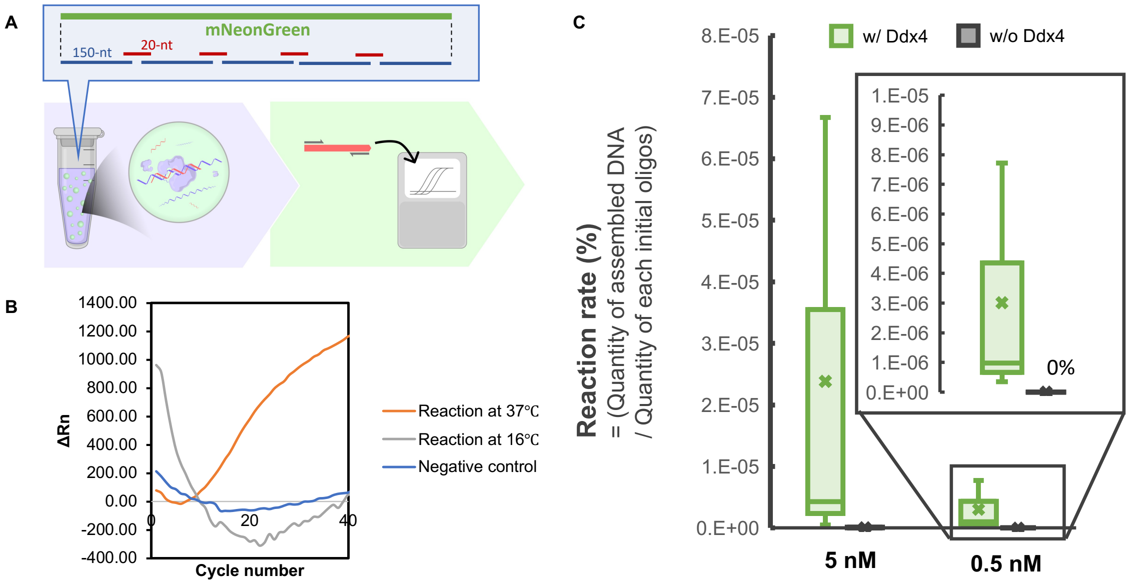

Supplementary Figure S7: qPCR results of oligo assembly towards protein coding DNA (**A**) Schematic image of the design of oligo assembly towards the protein coding DNA and experimental method of qPCR. (**B**) The qPCR experimental results of amplification curve. Red line shows the result of oligo assembly at 37℃. Gray line shows the results of oligo assembly at 16℃. Blue line shows the result of unassembled oligos. The oligo assembly was performed with 250 nM oligos without using Ddx4 phase separation. (**C**) The experimental qPCR result of oligo assembly towards protein coding DNA. The green bar shows the result with Ddx4 phase separation. The gray bar shows the result without Ddx4 phase separation. Using 5 nM oligos as the initial concentration, the average reaction rates, which can be calculated as quantity of assembled DNA per quantity of each initial oligos, are 2.38×10^{-5} % with Ddx4 and 4.12×10^{-8} % without Ddx4. Using 0.5 nM oligos as initial concentration, the average reaction rates are 3.01×10^{-6} % with Ddx4 and no valid signal meaning 0.00% without Ddx4.

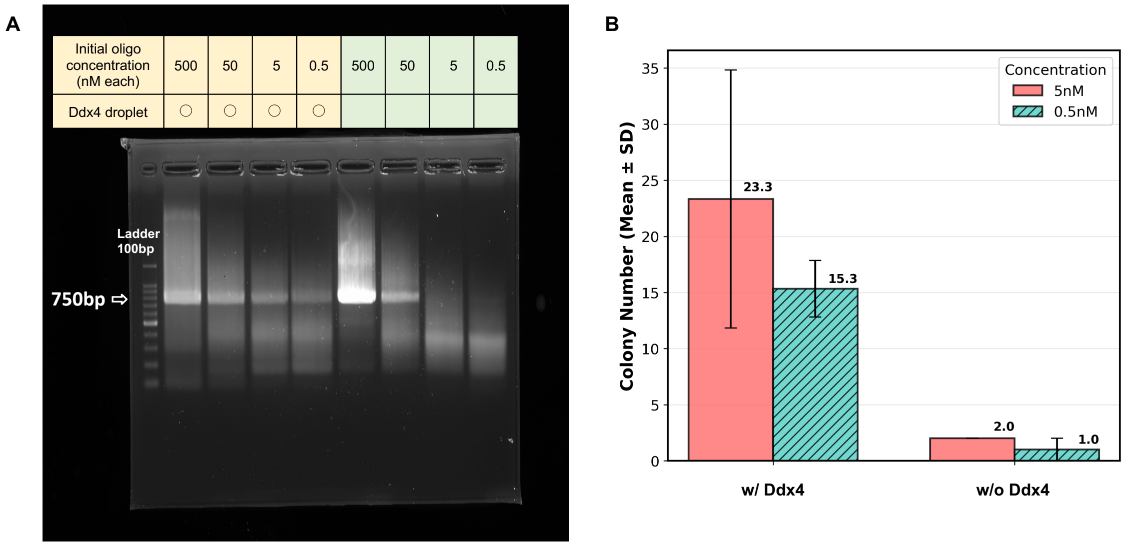

Supplementary Figure S8: PCR result of droplet enhanced oligo assembly (**A**) Result of Gel Electrophoresis after PCR amplification of assembled products. Left side of the gel image shows the result of oligo assembly using initial oligo concentration from 500 nM to 0.5 nM with Ddx4 phase separation. Right side of the gel image shows the result of oligo assembly using initial oligo concentration from 500 nM to 0.5 nM without Ddx4. The full length of assembled products was amplified with PCR amplification, and the desired size of the amplicon is 750 bp. (**B**) Number of transformed colonies with droplet enhanced assembly (w/ Ddx4) and with assembly w/o Ddx4 (w/o Ddx4) at 0.5 or 5.0 nM oligo mix. N=3.

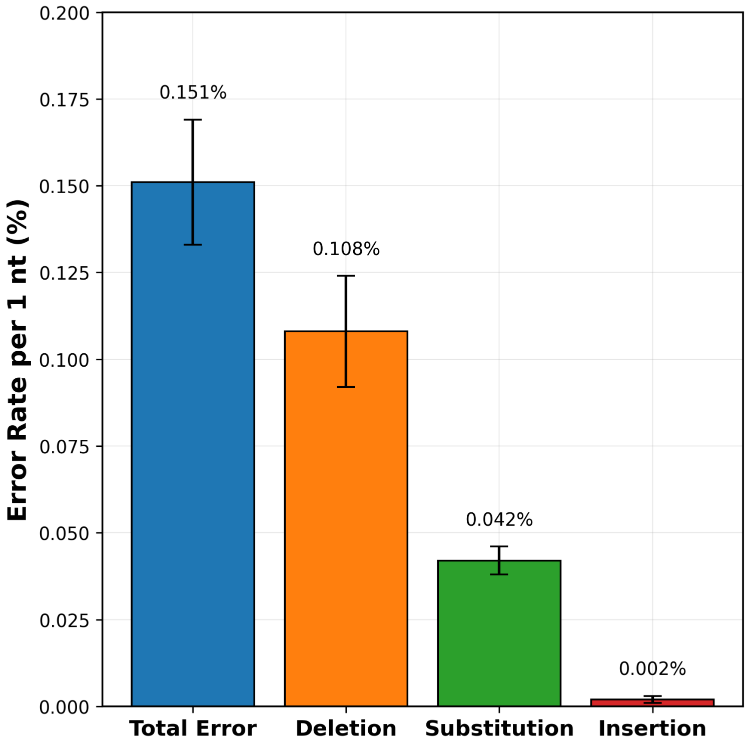

Supplementary Figure S9: Results of errors per nucleotide when NGS analysis is performed on synthetic oligos. The blue bars indicate the results when all error types are included. To the left of that is the result of classifying the errors by their respective error types. Error rate per nucleotide was calculated for each of the five oligos used in the analysis and then averaged based on n=5.

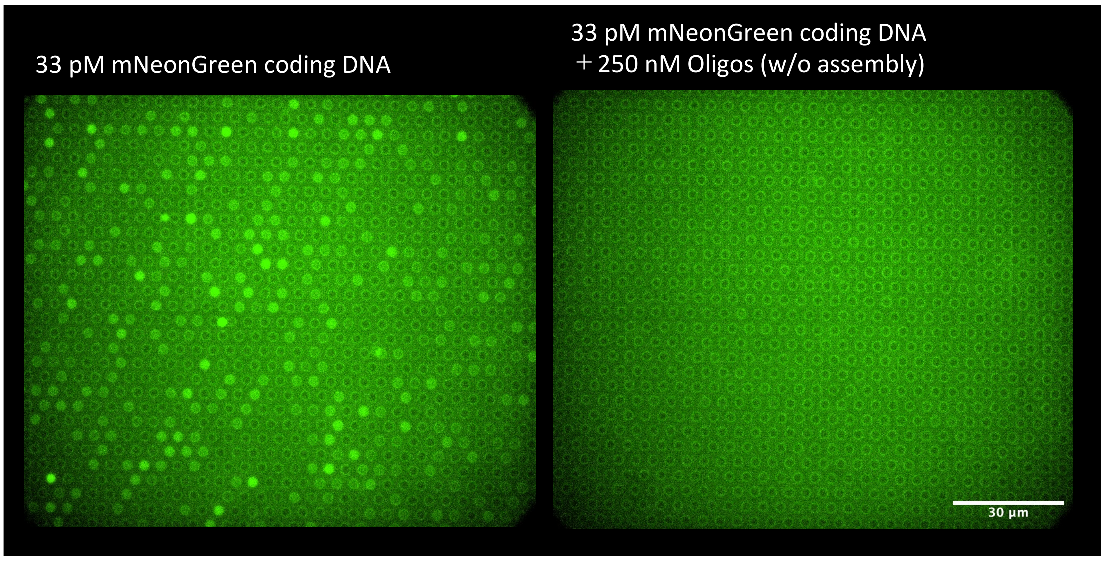

Supplementary Figure S10: The result after cell free expression on femto-litter reactor with or without un-assembled oligos. The left figure shows the result without un-assembled oligos, and the right figure shows the result with 250 nM un-assembled oligos. In this experiment, the mNeonGreen coding DNA was not attached on the femto-litter reactor but the DNA was only mixed with cell free expression solution and introduced into the reactor. The result of expression was observed by using fluorescent microscope.

A

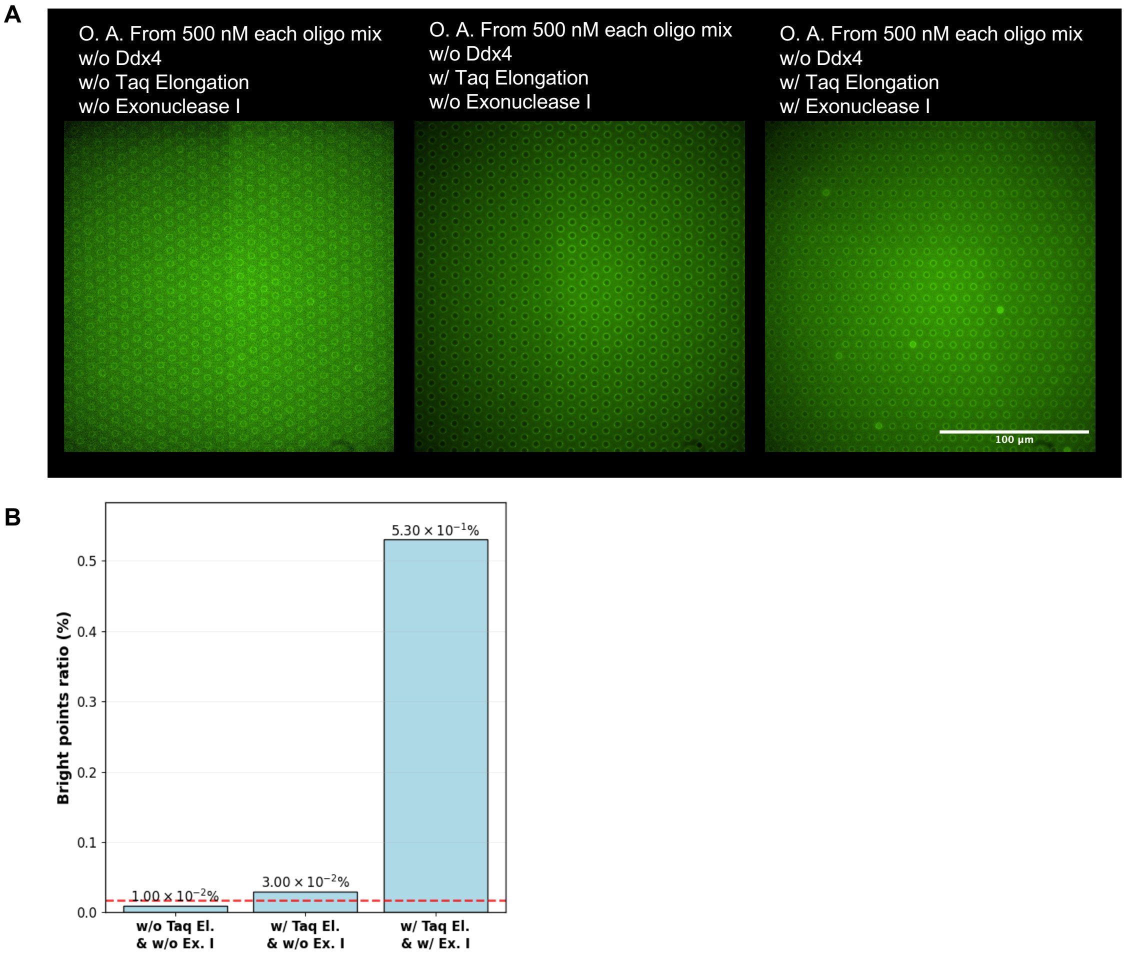

Supplementary Figure S11: Effect of processing for oligo assembled sample on the efficiency of cell free expression (**A**) The result image after cell free expression of assembled product with or without Taq Elongation and Exonuclease I process. These images were obtained by using fluorescent microscope. All results were obtained from assembled products using 500 nM oligos for initial concentration. In the leftmost case, where the Taq Elongation process is not performed, 10 μM of primers capable of duplicating the T7 promoter are added at the final concentration during cell-free protein expression. (**B**) The quantified result of (**A**). Y axis shows the bright points ratio = number of bright points / numbers of detected points. The dotted line shows the threshold of the result which is obtained from the result without using any DNAs.

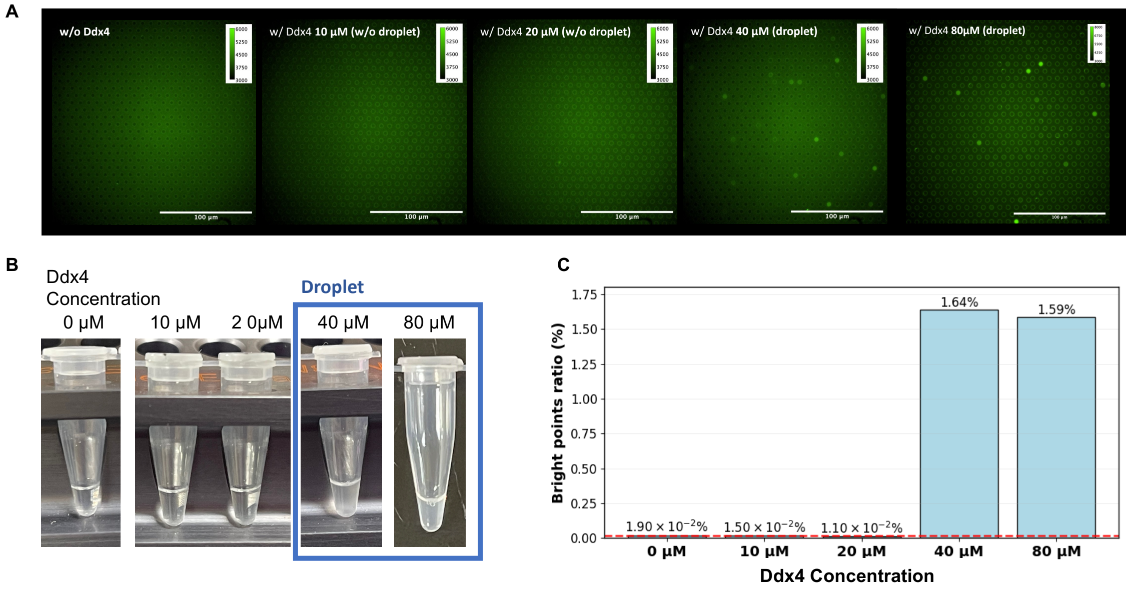

Supplementary Figure S12: Importance of phase separation (**A**) Fluorescent microscope image of femto-liter reactor after cell free expression showing the green fluorescence from mNeonGreen. The concentration of Ddx4 used at the oligo assembly step is different. The left side is the case without Ddx4, and the concentration used increases as one move to the right. Used Ddx4 concentrations are 0 μM, 10 μM, 20 μM, 40 μM and 80μM. The used initial oligo concentration is 50 nM for all conditions. (**B**) Photograph of the solution at the time of oligo-consolidation; only at 40 μM and 80μM a white cloudiness is observed, originating from the scattering of light by the phase-separated droplets. c) Quantitative result of protein expression from oligo assembly at various Ddx4 concentration. Only the data of 80μM is average value using n=3, which is the same result shown in Figure 5. Dotted line shows the threshold, in which the value is 1.79×10^{-2} %.

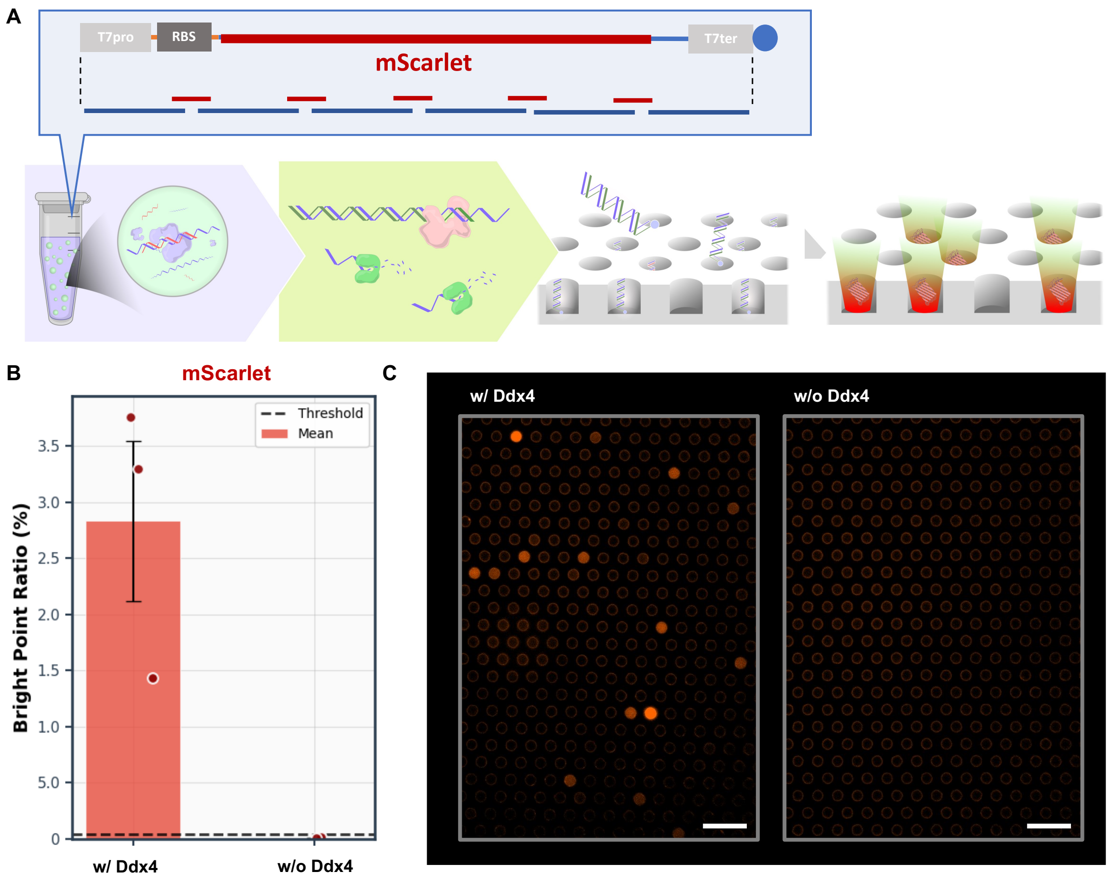

Supplementary Figure S13: Required time to confirm expression in the femto-liter reactor (**A**) The schematic image of cell-free protein prototyping integrating cell-free digital gene expression and droplet-enhanced oligo assembly. Initial oligo concentration was 1 nM. (**B**) Fraction of reactors expressing mScarlet protein with bright fluorescence signal (N=3). The threshold line is defined as mean+3 s. d. of the pseudo-positive signal (the bright reactor fraction in the absence of DNA at mScarlet channel); 3.91×10^{-2}. (**C**) Fluorescence microscope image of femto-liter reactors expressing mScarlet protein from droplet-enhanced assembly (left) and assembly w/o droplet (right).

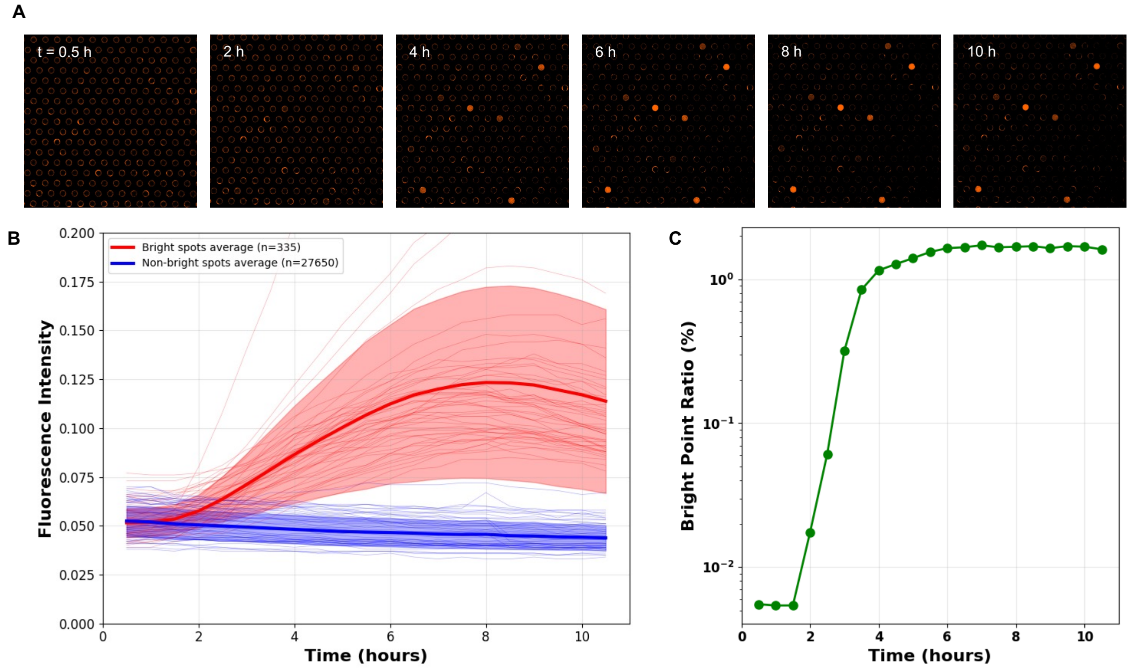

Supplementary Figure S14: Required time to confirm expression in the femto-liter reactor. (**A**) The timelapse image of cell free expression inside the femto-liter reactor. The used sample was obtained from oligo assembled products towards mScarlet coding DNA using phase separated droplets. The initial oligo concentration is 1 nM. Microscopic images were taken every 30 min at 96 visions while incubating at 30°C. A portion of the acquired data is used in the figure. (**B**) Time variation of the fluorescence intensity of the mScarlet channel in the reactors. The red color represents the result in the reactor that was finally counted as a bright spot, which is the average of 335 data. The light-colored area represents SD. The blue color represents the results of reactors that were finally determined not to be bright spots. This is not all the data, but the average of 50 randomly obtained data. The light-colored area represents SD. 8 hours or so later, the fluorescence intensity of the bright spots has reached a plateau. (**C**) Time variation of the percentage of reactors counted as bright spots, reaching a plateau after about 6 hours.

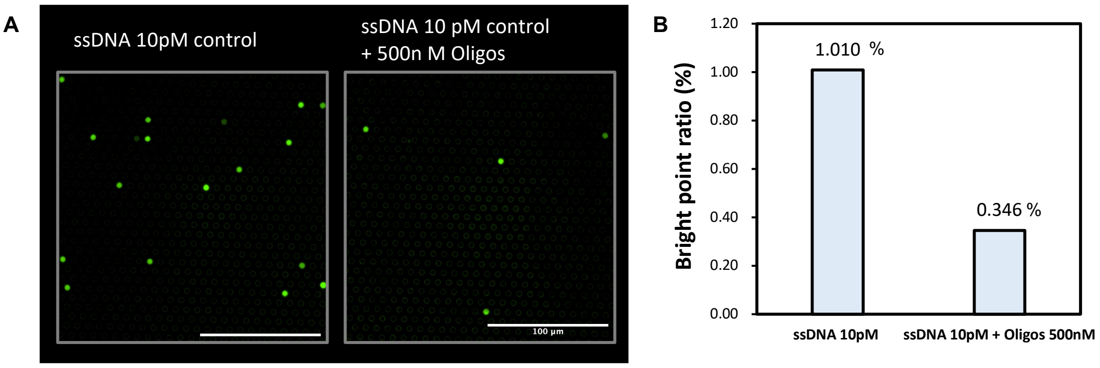

Supplementary Figure S15: Effect of residual unassembled oligos after the washing process. Biotinylated single-stranded DNA coding for mNeonGreen, with or without unassembled oligos, was extended to double-stranded DNA using Taq DNA polymerase. The dsDNA was treated with Exonuclease I and introduced into femto-liter reactors, where mNeonGreen was expressed.
(A) Representative images of the experiments with or without unassembled oligos. **(B)** Quantitative analysis of the results shown in **(A)**. The left and right bars represent the results in the absence and presence of unassembled oligos, respectively.

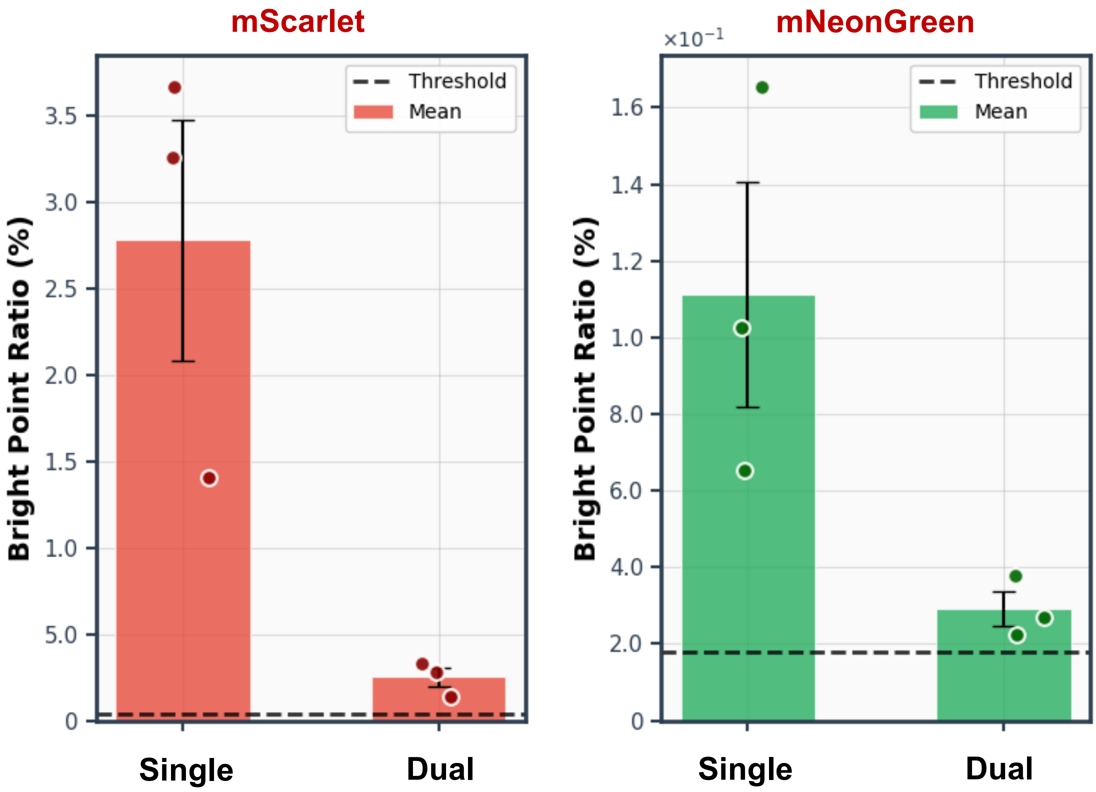

Supplementary Figure S16: Comparison of expression yields between single and dual expression. The data compare single expression (Fig. 5B and Supplementary Fig. S13B) and dual expression (Fig. 6B). Fractions of positive reactor found in mScarlet channel (red) or in mNeonGreen channel (green) (N=3). The initial oligo concentrations were 1 nM. Dotted lines represent the thresholds defined as mean+3 s.d. of pseudo-positive signal (the bright reactor fraction in the absence of DNA); the values are 3.91×10^{-2} for the mScarlet channel and 1.79×10^{-2} for the mNeonGreen channel.

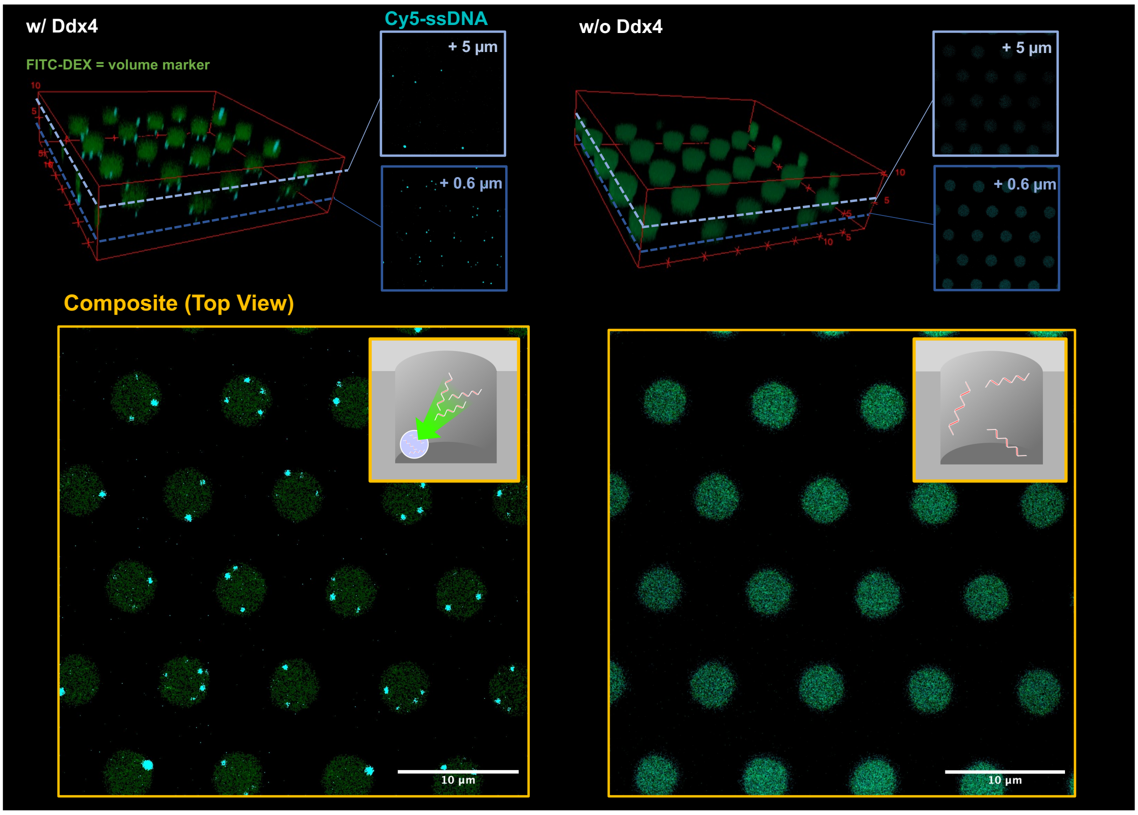

Supplementary Figure S17: Spatial distribution of Ddx4 droplets within microreactors. Upper panels show a 3D schematic of the microreactor alongside Cy5-ssDNA fluorescence images captured at heights of 0.5 μm and 3.0 μm from the reactor bottom. Lower panels show the composite top-view images of the reactors. The Ddx4 solution used was the upper phase obtained by centrifugation of a phase-separated mixture. Solutions with or without Ddx4, containing Cy5-labeled ssDNA (20 nt) and FITC-DEX, were loaded into the reactors. The solution also contains 1x T4 Ligase buffer (Takara). The used Ddx4 sample was The reactors were sealed with 30μL AE3000 (AGC) containing 0.1% (v/v) S386 (AGC) and 30μL Fomblin (Solvay). Droplet formation was triggered by an increase in Ddx4 concentration, resulting from water absorption into the sealing oil. Cyan and green fluorescence represent Cy5-ssDNA (1 μM) and FITC-DEX (0.1 % v/v), respectively. FITC-DEX was used as a non-accumulating volume indicator. In the presence of Ddx4, accumulated ssDNA was observed as small droplets within the reactor. In contrast, without Ddx4, ssDNA filled the entire reactor volume. These results indicate that Ddx4 droplets formed within the reactors and selectively

concentrated the ssDNA. The Cy5 channel intensities were corrected to ensure uniform brightness across the entire reactor.

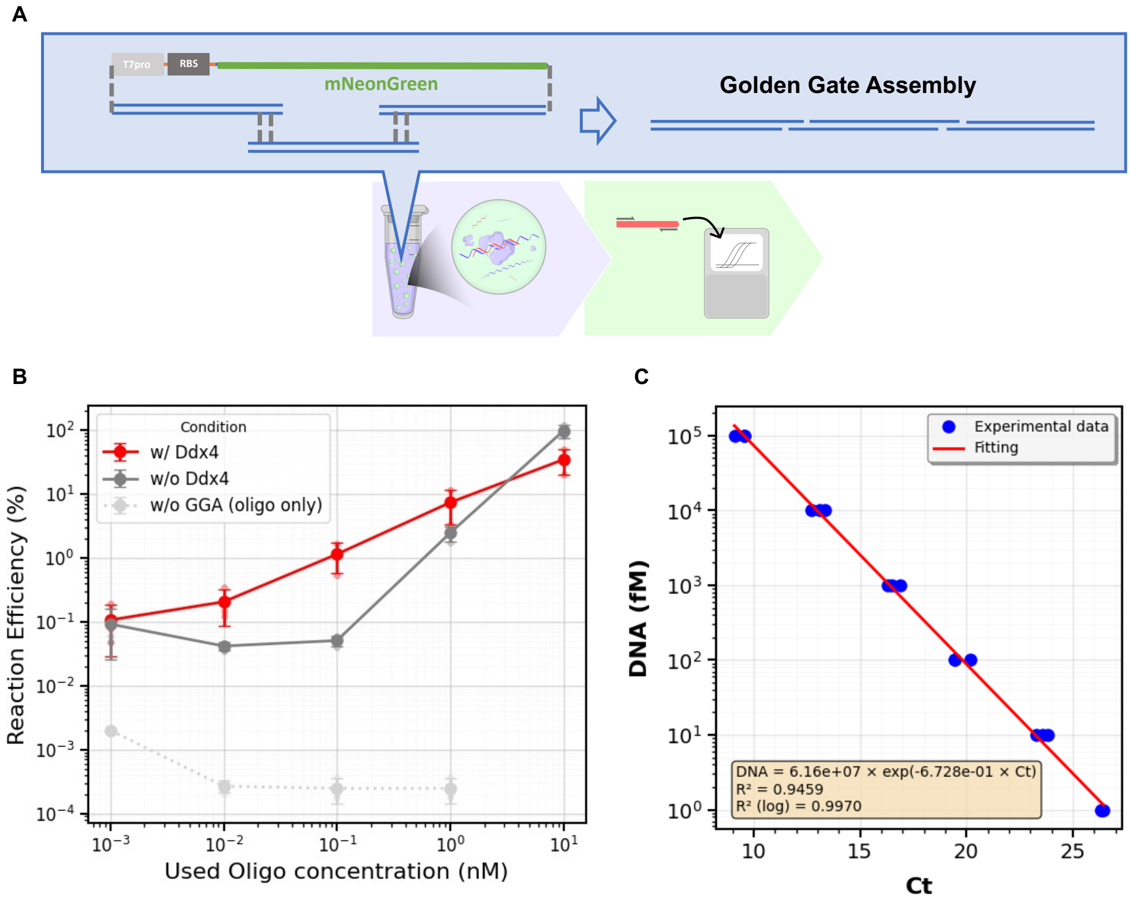

Supplementary Figure S18: qPCR results of Golden Gate Assembly. (**A**) Schematic image of the design of Golden Gate Assembly towards double stranded DNA and experimental method of qPCR. The used primer for qPCR can be annealed the end of the assembled product. (**B**) Reaction efficiency versus the initial oligo concentration. Reaction efficiency was defined as assembled DNA yield divided by theoretical maximum yield estimated from the initial oligo concentration. Golden Gate Assembly with Ddx4 droplet (Red), assembly without Ddx4 droplet (gray) and oligo without Golden Gate Assembly for negative control (light gray and dotted line). N=3 (**C**) Calibration curve for qPCR. DNA with all oligos assembled and duplex DNA encoding the same sequence were diluted in steps to obtain their respective Ct values. n=3 for each concentration. The experimental setup was shown in Supplementary Materials and Methods.

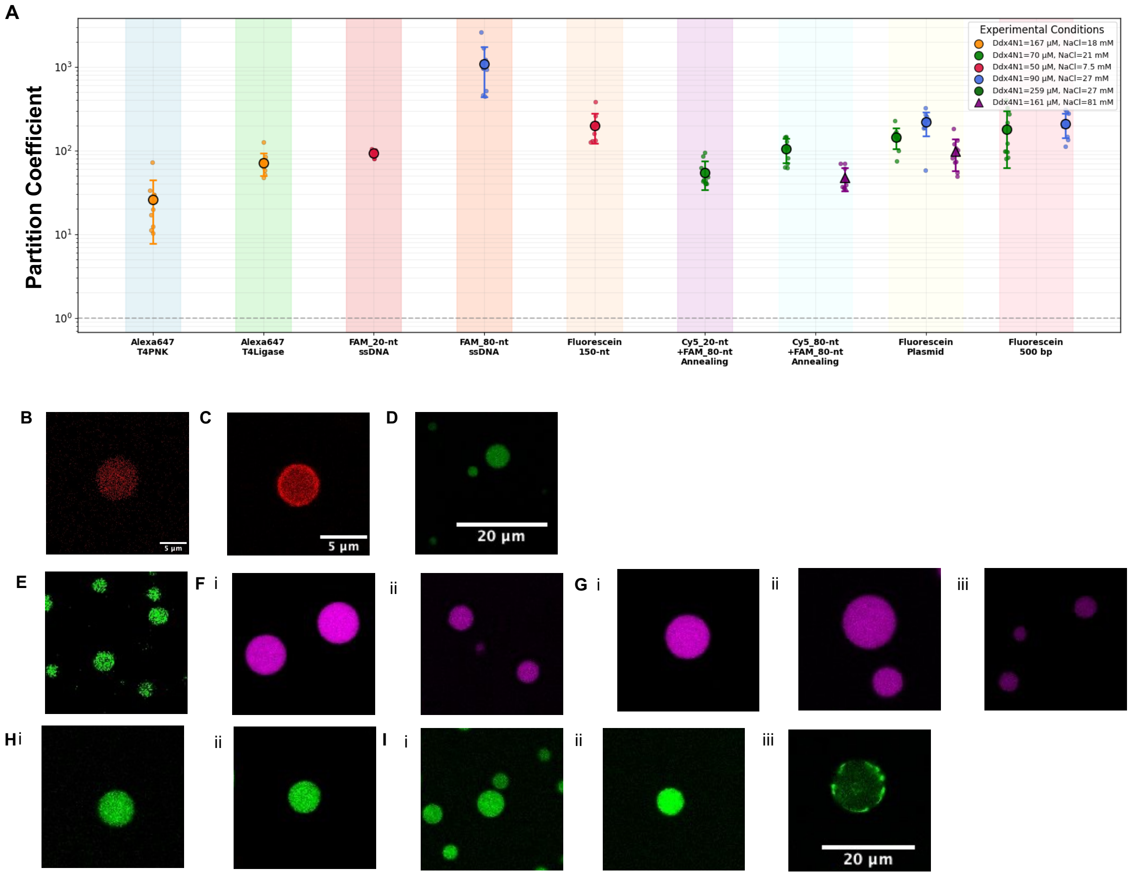

Supplementary Figure S19: Ddx4-mediated condensate formation under varying buffer conditions and nucleic acid substrates. (**A**) Quantitative analysis showing detailed buffer conditions and additional data corresponding to Figure 1. Circle markers represent results obtained at low NaCl concentrations (≤27 mM); triangle markers represent results at high NaCl concentration (81 mM). Each data point represents measurements from 10 individual droplets. The detailed enrichment factor was described in Supplementary Table S1. (**B-H**) Representative fluorescence microscopy images corresponding to all experimental conditions shown in panel. Each PCs was corrected based on FLIM experiments (Supplementary Figure S2). (**A**). All images were acquired using identical exposure settings. Individual panels show: (**B**) Condensate formation with Alexa 647-labeled T4 PNK. Experimental conditions: 167 µM Ddx4, 18 mM NaCl. (**C**) Condensate formation with Alexa 647-labeled T4 Ligase. Experimental conditions: 167 µM Ddx4, 18 mM NaCl. (**D**) Condensate formation with FAM-labeled 20-nt ssDNA (green fluorescence). Experimental conditions: 50 µM Ddx4, 7.5 mM NaCl. (**E**) Condensate formation with fluorescein-labeled 150-nt ssDNA (green fluorescence). Experimental conditions: 50 µM Ddx4, 7.5

mM NaCl. (**F**) Condensate formation with Cy5-labeled 20-nt ssDNA and FAM-labeled 80-nt ssDNA. Images show Cy5 signal. (**F**-i) 70 μM Ddx4, 21 mM NaCl. (**F**-ii) 259 μM Ddx4, 27 mM NaCl. (**G**) Condensate formation with Cy5-labeled 80-nt ssDNA and FAM-labeled 80-nt ssDNA. Images show Cy5 signal. (**G**-i) 70 μM Ddx4, 21 mM NaCl. (**G-**ii) 259 μM Ddx4N1, 27 mM NaCl. (**G**-iii) 161 μM Ddx4N1, 81 mM NaCl. (**H**) Condensate formation with fluorescein-labeled 500-bp dsDNA. (**H**-i) 70 μM Ddx4, 21 mM NaCl. (**H**-ii) 90 μM Ddx4, 27 mM NaCl. (**I**) Condensate formation with fluorescein-labeled 7569-bp plasmid DNA. (**I**-i) 70 μM Ddx4, 21 mM NaCl. (**I**-ii) 90 μM Ddx4, 27 mM NaCl. (**I**-iii) 161 μM Ddx4, 81 mM NaCl.

Conc. Ddx4^{N1} (μM)	Conc. NaCl (mM)	Used Fluorescent Labeled Substances	Mean Partition Coefficient
167	18	Alexa647_Ligase	71.8
167	18	Alexa647_PNK	26.1
50	7.5	Cy5_20nt	93.4
90	27	FAM_80nt	1093
50	7.5	Fluorescein 150nt	200
70	21	Cy5_20nt & FAM_80nt annealing FRET	37
259	27	Cy5_20nt & FAM_80nt annealing FRET	36.0
70	21	Cy5_80nt & FAM_80nt annealing FRET	19.1
259	27	Cy5_80nt & FAM_80nt annealing FRET	27.8
161	81	Cy5_80nt & FAM_80nt annealing FRET	16.7
70	21	Fluorescein Plasmid	145
90	27	Fluorescein Plasmid	219
161	81	Fluorescein Plasmid	97.4
70	21	Fluorescein 500bp	180
90	27	Fluorescein 500bp	210

Supplementary Table S1: Detailed Enrichment Factor at different condition

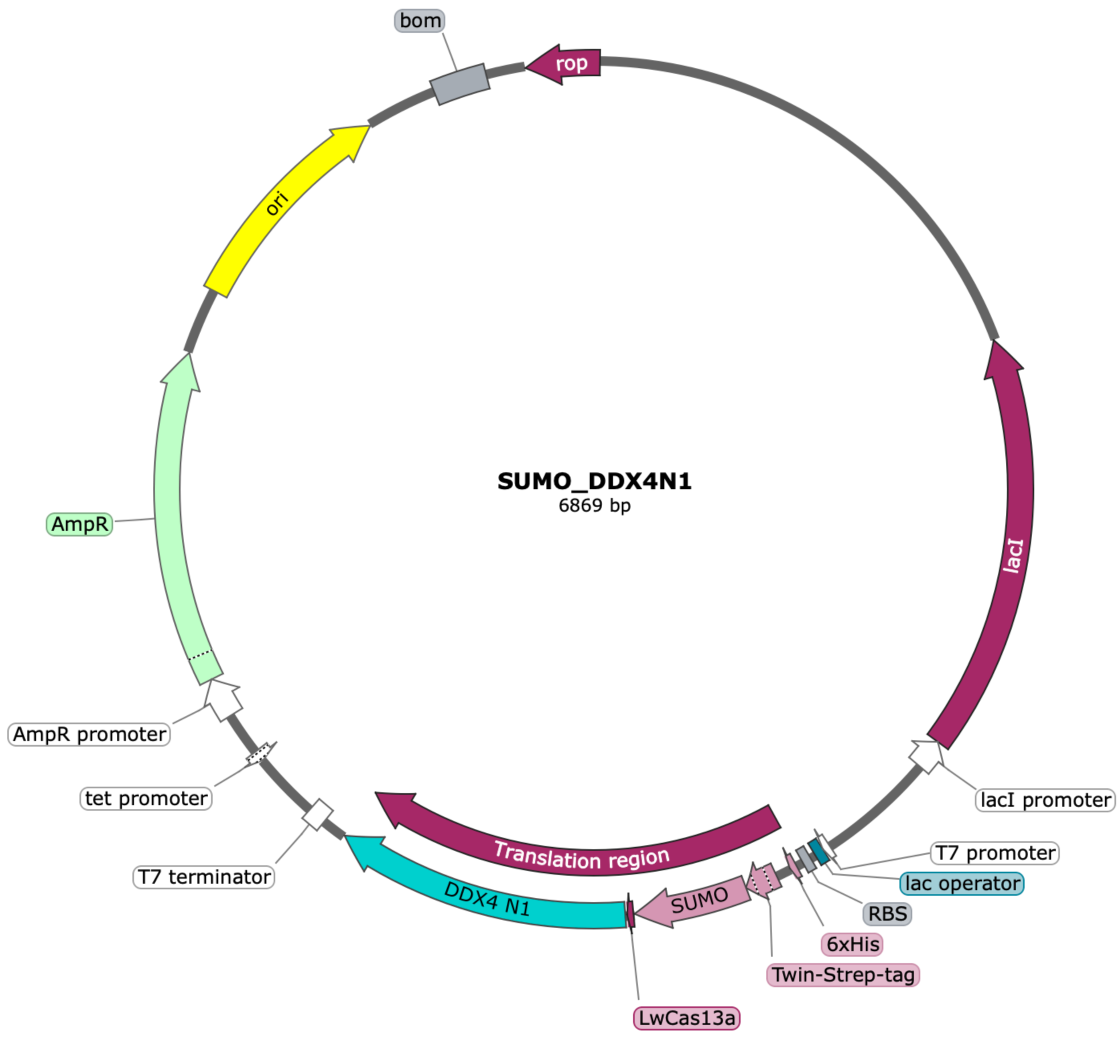

Supplementary Figure S20: Used Ddx4^{N1} plasmid. The used Ddx4^{N1} plasmid contained SUMO region, His tag (6xHis) and Twin-Strep-Tag in transcript region.

Supplementary Table S2: Protein Sequences

Protein Name	Amino Acid Sequence
TwinStrepTag+ SUMO+Ddx4[N1]	MGSSHHHHHHSSGLVPRGSHMASWSHPQFEKGGGSGGGSGGSAWSHP QFEKMSDSEVNQEAKPEVKPEVKPETHINLKVSDGSSEIFFKIKKTTPLRRL MEAFAKRQGKEMDSLRFLYDGIRIQADQTPEDLDMEDNDIIEAHREQIGGS MKVTKVDGSGAMGSMGDEDWEAEINPHMSSYVPIFEKDRYSGENGDNFN RTPASSSEMDDGPSRRDHFMKSGFASGRNFGNRDAGECNKRDNTSTMG GFGVGKSFGNRGFSNSRFEDGDSSGFWRESSNDCEDNPTRNRGFSKRG GYRDGNNSEASGPYRRGGRGSFRGCRGGFGLGSPNNDLDPDECMQRTG GLFGSRRPVLSGTGNGDTSQSRSGSGSERGGYKGLNEEVITGSGKNSWK SEAEGGESSDTQGPKVTKL*
Ddx4[N1]	SMKVTKVDGSGAMGSMGDEDWEAEINPHMSSYVPIFEKDRYSGENGDNF NRTPASSSEMDDGPSRRDHFMKSGFASGRNFGNRDAGECNKRDNTSTM GGFGVGKSFGNRGFSNSRFEDGDSSGFWRESSNDCEDNPTRNRGFSKR GGYRDGNNSEASGPYRRGGRGSFRGCRGGFGLGSPNNDLDPDECMQRT GGLFGSRRPVLSGTGNGDTSQSRSGSGSERGGYKGLNEEVITGSGKNSW KSEAEGGESSDTQGPKVTKL*

Supplementary Table S3: Oligonucleotide Sequences

FRET Analysis Oligonucleotides

Name	Sequence (5' to 3')
20nt-5'FAM	[FAM] CTTGGTCACTTTCATGGATC
20nt-5'Cy5-noannealing	[FAM] CTTGGTCACTTTCATGGATC
80nt-5'Cy5	CTTGGTCACTTTCATGGATCCACCAATCTGTTCTCTGTGAGCCTCAA TAATATCGTTATCCTCCATGTCCAAATCTTCAG [Cy5]
80nt-3'FAM	[FAM] CTGAAGATTTGGACATGGAGGATAACGATATTATTGAGGCTCACAGA GAACAGATTGGTGGATCCATGAAAGTGACCAAG

Oligo Assembly System 1 (9-oligos)

Template Oligos (P1-P5)	Sequence (5' to 3')
P1	CGCGGAACCCCTATTTGTTTATTTTTCTAAATACATTCAAATAT GTATCCGCTCATGAGACAATAACCCTGATAAATGCT
P2	TCAATAATATTGAAAAAGGAAGAGTATGAGTATTCAACATTTCC GTGTCGCCCTTATTCCCTTTTTTGCGGCATTTTGCC
P3	TTCCTGTTTTTGCTCACCCAGAAACGCTGGTGAAAGTAAAAGA TGCTGAAGATCAGTTGGGTGCACGAGTGGGTTACATC
P4	GAACTGGATCTCAACAGCGGTAAGATCCTTGAGAGTTTTCGCC CCGAAGAACGTTTTCCAATGATGAGCACTTTTAAAGT
P5	TGTGAAATTAATACGACTCACTATAGGGAGACCACAACGGTTT CCCTCTAGAAATAATTTTGTTTAACTTTAAGAAGGAG

Linker Oligos (L1-L4)	Sequence (5' to 3')
L1	TATTATTGAAGCATTTATCA
L2	AAAACAGGAAGGCAAAATGC
L3	GATCCAGTTCGATGTAACCC
L4	ACATAGCAGAACTTTAAAAG

qPCR Primers and Probes

Name	Sequence (5' to 3')
Primer-forward	CATTCTGAGAATAGTGTATGCGGCGACCGAGTTGCTCTTGCCCG GCGTCAACACGGGATAATACCGCGCCACATAGCAGA
Primer-reverse	CATTCTGAGAATAGTGTATGCGGCGACCGAGTTGCTCTTGCCCG GCGTCAACACGGGATAATACCGCGCCACATAGCAGA
TaqMan probe	[FAM]- ATTCCCTTTTTTGCGGCATTTTGCCTTCCTGTTTTTGCTCACCCAG AAAC-[BHQ1]

Linker Oligos (L1-L5-mNG)	Sequence (5' to 3')
L1-mNG	CCGTTGATGGAGCCAAAGAT
L2-mNG	TAGGGCAGGTACTGATGGAA
L3-mNG	ACCTGGGCCTCTCCTTTGAT
L4-mNG	TAGCGCTTGCCATTTCCAGT
L5-mNG	TCGGTAAAGGCCTTTTGCCA

Oligo Assembly System 2 (9-oligos)

Template Oligos (P1-P5-mNG)	Sequence (5' to 3')
P1-mNG	ATGTCTAAAATAAAAGTGAGCAAGGGCGAGGAGGATAACATGGC CTCTCTCCCAGCGACACATGAGTTACACATCTTTGGCTCCATCAA CGGTGTGGACTTTGACATGGTGGGTCAGGGCACCGGCAATCCAA ATGATGGTTATGAGGAG
P2-mNG	TTAAACCTGAAGTCCACCAAGGGTGACCTCCAGTTCTCCCCCTGG ATTCTGGTCCCTCATATCGGGTATGGCTTCCATCAGTACCTGCCC TACCCTGACGGGATGTCGCCTTTCCAGGCCGCCATGGTAGATGG CTCCGGATACCAAGTC
P3-mNG	CATCGCACAATGCAGTTTGAAGATGGTGCCTCCCTTACTGTTAAC TACCGCTACACCTACGAGGGAAGCCACATCAAAGGAGAGGCCCA GGTGAAGGGGACTGGTTTCCCTGCTGACGGTCCTGTGATGACCA ACTCGCTGACCGCTGCG
P4-mNG	GACTGGTGCAGGTCGAAGAAGACTTACCCCAACGACAAAACCAT CATCAGTACCTTTAAGTGGAGTTACACCACTGGAAATGGCAAGCG CTACCGGAGCACTGCGCGGACCACCTACACCTTTGCCAAGCCAA TGGCGGCTAACTATCTG
P5-mNG	AAGAACCAGCCGATGTACGTGTTCCGTAAGACGGAGCTCAAGCA CTCCAAGACCGAGCTCAACTTCAAGGAGTGGCAAAAGGCCTTTAC CGATGTGATGGGCATGGACGAGCTGTACAAGTAACACGATTTTGA CTTCCTAGCATAACCC

Additional Oligonucleotides	Sequence (5' to 3')
Primer-forward-mNG	ATGTCTAAAATAAAAGTGAGCAAGGGCGAGGAGGATAAC
Primer-reverse-mNG	GGGTTATGCTAGGAAGTCAAAATCGTGTTACTTGTACAGC
TaqMan probe-mNG	[FAM]-CGCTGACCGCTGCGGACTGG-[BHQ1]

Oligo Assembly System 3 (11-oligos)

Template Oligos (P1-P6-mNG)	Sequence (5' to 3')
P1-mNG_Ex	GAGCCAAAGATGTGTAACTCATGTGTCGCTGGGAGAGAGGCCATGTTATCCTCCTCGCCCTTGCTCACTTTTATTTTAGACATAGCTCTCCTTCTTAAAGTTAAACAAACCGCTCACAATTCCCCTATAGTGAGTCGTATTAATTTCGCG
P2-mNG_Ex	TACTGATGGAAGCCATACCCGATATGAGGGACCAGAATCCAGGGGGAGAACTGGAGGTCACCCTTGGTGGACTTCAGGTTTAACTCCTCATAACCATCATTTGGATTGCCGGTGCCCTGACCCACCATGTCAAAGTCCACACCGTTGATG
P3-mNG_Ex	TCTCCTTTGATGTGGCTTCCCTCGTAGGTGTAGCGGTAGTTAACAGTAAGGGAGGCACCATCTTCAAACTGCATTGTGCGATGGACTTGGTATCCGGAGCCATCTACCATGGCGGCCTGGAAAGGCGACATCCCGTCAGGGTAGGGCAGG
P4-mNG_Ex	CCATTTCCAGTGGTGTAACTCCACTTAAAGGTACTGATGATGGTTTTGTCGTTGGGGTAAGTCTTCTTCGACCTGCACCAGTCCGCAGCGGTCAGCGAGTTGGTCATCACAGGACCGTCAGCAGGGAAACCAGTCCCCTTCACCTGGGCC
P5-mNG_Ex	GCCTTTTGCCACTCCTTGAAGTTGAGCTCGGTCTTGGAGTGCTTGAGCTCCGTCTTACGGAACACGTACATCGGCTGGTTCTTCAGATAGTTAGCCGCCATTGGCTTGGCAAAGGTGTAGGTGGTCCGCGCAGTGCTCCGGTAGCGCTTG

Template Oligos (P1-P6-mNG)	Sequence (5' to 3')
P6-mNG_Ex	TCAGCAAAAAACCCCTCAAGACCCGTTTAGAGGCCCCAAGGGGT TATGCTAGGAAGTCAAAATCGTGTTACTTGTACAGCTCGTCCATG CCCATCACATCGGTAAAG [Biotin]

Primer	Sequence (5' to 3')
Extension Primer	CGCGAAATTAATACGACTCACTATAGGGGAATTGTGAGC

Linker Oligos (20 nt)	Sequence (5' to 3')
L1-mNG_Ex	CCGTTGATGGAGCCAAAGAT
L2-mNG_Ex	TAGGGCAGGTACTGATGGAA
L3-mNG_Ex	ACCTGGGCCTCTCCTTTGAT
L4-mNG_Ex	TAGCGCTTGCCATTTCCAGT
L5-mNG_Ex	TCGGTAAAGGCCTTTTGCCA

Oligo Assembly System 4 (11-oligos)

Template Oligos (P1-P6-mNG)	Sequence (5' to 3')
P1-mSc_Ex	TTCATAGATCCCTCCATGTGAACTTTGAAGCGCATAAACTCCTTAA TCACGGCTTCCCCTTTGCTAACTTTTATTTTAGACATAGCTCTCCT TCTTAAAGTTAAACAAACCGCTCACAATTCCCCTATAGTGAGTCGT ATTAATTTCGCG
P2-mSc_Ex	ATAAACGCCCGAGAACCATACATGAACTGCGGACTCAGAATATCC CAGGAAAACGGAAGTGGCCCGCCTTTAGTCACCTTCAGTTTCGCT GTCTGAGTGCCTTCATAGGGACGACCTTCACCTTCGCCTTCAATC TCGAACTCATGACCG

Template Oligos (P1-P6-mNG)	Sequence (5' to 3')
P3-mSc_Ex	TTCACTTTGTAGATCAGCGTTCCATCCTCCAAGCTCGTGTCTTGGGTAACGGTTACGGCACCACCATCCTCGAAGTTCATGACACGTTCCCACTTAAAGCCTTCCGGAAAGCTCTGTTTATAGTAGTCCGGAATATCCGCGGGATGTTTG
P4-mSc_Ex	TAGCGACCGCCATCTTTCAGACGTAAAGCCATTTTGATGTCCCCTTTCAGAACGCCATCTTCCGGATAGAGGCGTTCGGTCGATGCTTCCCAACCCATGGTTTTCTTCTGCATCACCGGACCGTCTGGTGGAAAGTTCGTACCGCGTAAT
P5-mSc_Ex	GTGGAATGCCGGCCTTCTGAGCGTTCATACTGTTCGACCACGGTATAATCCTCATTGTGACTCGTAATGTCCAGTTTGCGATCGACATTGTATGCGCCAGGCATTTGCACCGGTTTCTTGGCTTTGTAGGTGGTTTTGAAATCCGCCAGA
P6-mSc_Ex	TTCAGTCAGCAAAAAACCCCTCAAGACCCGTTTAGAGGCCCCAAGGGGTTATGCTAGGAAGTCAAAATCGTGTTACTTGTACAGTTCGTCCATGCCTCCT [Biotin]

Linker Oligos (20 nt)	Sequence (5' to 3')
L1-mSc_Ex	GATCTATGAACGGTCATGAG
L2-mSc_Ex	GGGCGTTTATCAAACATCCC
L3-mSc_Ex	ACAAAGTGAAATTACGCGGT
L4-mSc_Ex	GCGGTCGCTATCTGGCGGAT
L5-mSc_Ex	GGCATTCCACAGGAGGCATG

Oligo Assembly System 5 (3-oligos)

GGA PCR Fragments (~300nt)	Sequence (5' to 3')
mNG_BsmI_1	TAATACGACTCACTATAGGGATAATACAACGGTTTTTGTTTAACTTTAAGAA GGAGATATACATATGGTTTCCAAAGGTGAGGAAGACAACATGGCATCGTT ACCAGCAACCCACGAATTGCACATTTTCGGCAGCATCAACGGGGTGGACT TTGACATGGTAGGGCAAGGTACGGGTAATCCGAACGACGGTTACGAAGA ACTTAACTTAAAGTCGACAAAAGGCGATCTCCAATTCAGCCCATGGATATT AGTGCCGCACATGAGACCTAAGACGCTTGGAATTACAGGTATCCG
mNG_BsmI_2	AGGTCTCACACATCGGCTATGGATTCCATCAGTATCTGCCATACCCCGAC GGCATGAGTCCATTCCAAGCGGCTATGGTCGATGGCTCAGGGTACCAAG TGCACCGCACGATGCAATTTGAGGATGGTGCGTCGCTTACAGTGAATTAT AGATACACTTACGAGGGTTCACACATTAAGGGCGAAGCTCAGGTTAAAGG CACGGGATTCCCCGCAGACGGGCCTGTTATGACCAATAGCCTGACCGCG GCCGACTGGTGCCGCAGTAAGAtGAGACGtAAGACGCTTGGAATTACAGG TATCCG
mNG_BsmI_3	AGGTCTCAAAGAAGACTTATCCTAACGACAAAACTATTATAAGCACATTTA AATGGAGCTACACCACCGGCAATGGTAAGCGCTATCGTAGCACGGCCCG TACAACCTACACGTTCGCCAAACCTATGGCAGCGAACTATCTGAAGAATC AACCGATGTACGTATTTCGTAAGACCGAGCTTAAGCATTCAAAGACTGAAC TCAATTTTAAGGAATGGCAGAAGGCTTTCACAGATGTCATGGGTATGGAC GAACTGTATAAGGGTGGCAGTCTGGTTTAAGACGCTTGGAATTACAGGTA TCCG

PCR primer	Sequence (5' to 3')
GA_qPCR_F	ATGGTTTCCAAAGGTGAGGAAGACAACATGGC
GA_qPCR_R	TTATACAGTTCGTCCATACCCATGACATCTGTGAAAGC

Supplementary Table S4: Example of estimated cost of genes using oligo pools or direct gene synthesis from vendors

Vendor	oligopool size	Parallelization level or 1kbp gene	Cost	Cost per gene
**IDT**	200nt x 5 x 1 = 1,000 base	1 gene	¥13,400 JPY / Pool	≈ $86.5 USD
	200nt x 5 x 2 = 2,000 base	2 gene	¥13,400 JPY / Pool	≈ $43.2 USD
	200 nt x 5 x 10 = 10,000 base	10 genes	¥3 JPY / Base	≈ $19.4 USD
	200 nt x 5 x 100 = 100,000 base	100 genes	¥2 JPY / Base	≈ $12.9 USD
	200 nt x 5 x 1,000 = 1,000,000 base	1,000 genes	¥1 JPY / Base	≈ $6.45 USD
	200 nt x 5 x 10,000 = 10,000,000 base	10,000 genes	¥1 JPY / Base	≈ $6.45 USD
**GenScript**	167 nt x 6 oligos	1 gene	¥233,600 JPY / Pool	≈ $1,507 USD
	167 nt x 12 oligos	2 gene	¥233,600 JPY / Pool	≈ $754 USD
	167 nt x 60 oligos	10 genes	¥233,600 JPY / Pool	≈ $151 USD
	167 nt x 600 oligos	100 genes	¥233,600 JPY / Pool	≈ $15.1 USD
	167 nt x 6,000 oligos	1,000 genes	¥387,200 JPY / Pool	≈ $2.50 USD

	167 nt x 60,000 oligos	10,000 genes	¥844,800 JPY / Pool	≈ $0.545 USD
**Eurofins (Gene fragments)**	1,000 bp	1 genes	$170 USD	$170 USD
	1,000 bp x 10,000	10,000 genes	$1,700,000 USD	$170 USD

Used rate is 1 USD = 155 JPY. These data were based on the websites of venders. (2-4)

Supplementary References

1. Lumiprobe Corporation. Fluorophores. https://www.lumiprobe.com/t/fluorophores (accessed Dec 10, 2025).

2. IDT Corporation. oPools Oligo Pools. https://sg.idtdna.com/pages/products/custom-dna-rna/dna-oligos/custom-dna-oligos/opools-oligo-pools (accessed Dec 10, 2025)

3. Gen Script Corporation. GenTitan™ Oligo pool synthesis.

https://www.genscript.jp/oligo-pools.html (accessed Dec 10, 2025)

4. Eurofins Genomics Corporation. Gene Fragments for Gene Synthesis | Eurofins Genomics. https://eurofinsgenomics.com/en/products/gene-synthesis/genestrands/ (accessed Dec 24, 2025)
